# Effectiveness of digital health interventions for asthma: a systematic review and meta-analysis

**DOI:** 10.3389/fpubh.2026.1910816

**Published:** 2026-08-05

**Authors:** Chongxiao Ning, Xunxun Yuan, Zhenzhen Feng, Jiansheng Li, Jiajia Wang

**Affiliations:** 1 Lung Disease Diagnosis and Treatment Center, National Medical Center, The First Affiliated Hospital of Henan University of Chinese Medicine, Zhengzhou, China; 2 The First Clinical Medical College, Henan University of Chinese Medicine, Zhengzhou, China; 3 Collaborative Innovation Center for Chinese Medicine and Respiratory Diseases Co-Constructed by Henan Province & Education Ministry of P.R. China, Henan Key Laboratory of Chinese Medicine for Respiratory Diseases, Henan University of Chinese Medicine, Zhengzhou, China

**Keywords:** asthma, meta-analysis, mHealth, mobile health, self-management, systematic review

## Abstract

**Background:**

Asthma, a prevalent chronic respiratory condition, frequently leads to issues like inadequate symptom management, diminished quality of life, and reduced lung function among patients. Proficient self-care plays a pivotal role in symptom control and enhancing quality of life. Digital health interventions have shown promise in enhancing treatment outcomes, mitigating acute exacerbations, and elevating patients' quality of life by enhancing self-management skills.

**Objective:**

This systematic review and meta-analysis aimed to assess the clinical impact of digital health interventions on asthma management, encompassing asthma control [evaluated using the Asthma Control Test (ACT) and Asthma Control Questionnaire (ACQ)], quality of life [assessed through the Asthma Quality of Life Questionnaire (AQLQ), Mini Asthma Quality of Life Questionnaire (mini-AQLQ), and Pediatric Asthma Quality of Life Questionnaire (PAQLQ)], pulmonary function [including forced expiratory volume in 1 second (FEV_1_), FEV_1_/forced vital capacity (FVC), and FEV_1_% predicted], and health care use indicators (measured by the frequency of hospitalizations and emergency visits).

**Methods:**

This study used preset search terms to systematically search PubMed, Embase, Cochrane, Scopus, and Web of Science databases until December 31, 2025. Randomized controlled trials on digital health interventions for asthma were screened. Two reviewers independently completed literature screening, data extraction, and bias risk assessment. The results covered asthma control, quality of life, lung function, and health care use indicators.

**Results:**

This analysis included 24 randomized controlled trials (RCTs) involving 5,155 participants across nine countries. Among these trials, 18 involved medical professionals utilizing digital platform applications, while two focused on compliance strategies. The digital tools for asthma primarily emphasize self-management systems, which encompass self-monitoring, medication reminders, and the dissemination of health information. The meta-analysis results indicated that digital health interventions significantly improved overall ACT scores [standardized mean difference (SMD) = 0.43, 95% CI: 0.35 to 0.51, *P* < 0.001]. Significant improvements were also observed at the 3-month follow-up (SMD = 0.45, 95% CI: 0.27 to 0.62, *P* < 0.001), 6-month follow-up (SMD = 0.41, 95% CI: 0.29 to 0.52, *P* < 0.001), and 12-month follow-up (SMD = 0.35, 95% CI: 0.04 to 0.65, *P* = 0.025). Similarly, a significant overall reduction in ACQ scores was associated with digital health interventions (SMD = −0.37, 95% CI: −0.66 to −0.08, *P* = 0.013), driven primarily by the 12-month follow-up subgroup (SMD = −0.48, 95% CI: −0.82 to −0.13, *P* = 0.007). In terms of quality of life, digital health intervention can significantly improve the quality of life of patients, overall AQLQ score (SMD = 0.98, 95% CI: 0.49 to 1.47, *P* < 0.001), 3 months (SMD = 0.55, 95% CI: 0.23 to 0.86, *P* = 0.001). At 6 months (SMD = 1.41, 95% CI: 1.06 to 1.75, *P* < 0.001) and 12 months follow-up (SMD = 0.60, 95% CI: 0.35 to 0.85, *P* < 0.001); the mini-AQLQ score exhibited a modest improvement (SMD = 0.20, 95% CI: 0.06 to 0.34, *P* = 0.005) at 3 months (SMD = 0.29, 95% CI: 0.02 to 0.55, *P* = 0.034) and at 12 months (SMD = 0.22, 95% CI: 0.03 to 0.40, *P* = 0.023). Nevertheless, no significant differences were observed in the Pediatric Asthma Quality of Life Questionnaire, lung function, emergency treatment, or hospitalization risk.

**Conclusion:**

Our study suggests that digital health interventions may benefit patient-reported asthma control and quality of life, but have no significant effect on objective physiological indicators or acute healthcare utilization. In the future, high-quality, large-sample and long-term randomized controlled trials are needed to further verify these findings.

**Systematic Review Registration:**

identifier PROSPERO CRD420251076990, URL: https://www.crd.york.ac.uk/PROSPERO/view/CRD420251076990.

## Introduction

Asthma is a heterogeneous disease marked by chronic airway inflammation and hyperresponsiveness (1). Research indicates that approximately 300 million individuals globally are affected by asthma, with only 44.1% of children, 55.4% of adolescents, and 61.1% of adults achieving adequate control of their condition. This situation is characterized by a high incidence rate, low control rates, and a diminished quality of life (2, 3). The challenges of poor asthma control and reduced quality of life have emerged as pressing clinical concerns that necessitate effective solutions (4). Effective self-management plays a crucial role in the prevention and treatment of asthma, being vital for symptom control and enhancing quality of life (5–7). There has been a growing interest in the potential of digital tools for asthma self-management in recent years. Evidence suggests that digital health interventions can enhance the quality of life and symptom control for asthma patients by facilitating self-management, enabling continuous medication monitoring, and improving treatment adherence (8–10).

Despite advances in the development of digital interventions for asthma self-management, existing studies on digital interventions for asthma have primarily focused on a single technical domain (e.g., basic remote monitoring) or yielded inconsistent results (11, 12). These limitations underscore the importance of conducting rigorous systematic reviews and meta-analyses, incorporating the most recent evidence, evaluating the efficacy of digital tools, and accounting for variations in intervention types and participant characteristics. In this context, our study aims to comprehensively evaluate digital health interventions for asthma management by integrating patient-centered efficacy indicators with healthcare resource utilization data. The study assesses the potential of existing digital health tools to enhance asthma control, improve patients' quality of life, enhance pulmonary function, and reduce acute medical service utilization, elucidating the clinical value of digital health intervention technologies.

## Methods

This systematic review and meta-analysis strictly follows the Systematic Review and Meta-Analysis Report Items (PRISMA) guidelines (13). The research protocol has been registered in the International System Review Prospective Registry (CRD420251076990).

### Search strategy

As of December 31, 2025, PubMed, Embase, Cochrane, Web of Science, and Scopus databases were systematically searched to screen relevant literature. The retrieval uses a combination of index terms such as medical subject words (MeSH) or Emtree, and matches them with the retrieval field. The following keywords were included: “Asthma OR Asthmas OR Asthma, Bronchial OR Bronchial Asthma OR asthma bronchiale OR asthma pulmonale OR asthmatic OR asthmatic subject OR bronchus asthma OR chronic asthma OR lung allergy” AND “Telemedicine OR Tele^*^ OR Mobile Health OR mHealth OR eHealth OR biosensor OR remote monitoring OR Internet OR Smartphone^*^ OR Phone^*^ OR Mobile Application^*^ OR App OR Web OR website^*^ OR digital health OR online OR information technology” AND “self-management OR self care OR self management OR self monitoring OR self-monitoring OR self-care” AND “randomized controlled trial OR random^*^ controlled trial OR controlled clinical trial^*^ OR random^*^ OR trial^*^.” The complete retrieval strategy of each database is detailed in Supplementary File 2. In addition, we conducted manual searches by reviewing the reference lists of relevant topic reviews and screening out eligible articles.

### Eligibility criteria

The specific inclusion criteria are as follows: (1) Subjects must be patients diagnosed with asthma; (2) Interventions must be conducted via a network-based remote digital health management platform; (3) The control group should receive usual care interventions; (4) Assessment indicators must encompass the effects of interventions on overall health or at least one related health indicator, such as asthma control, quality of life, lung function, and utilization of medical resources; (5) The study design must be a randomized controlled trial (RCT). Since the inclusion of unpublished studies may introduce bias, this systematic review and meta-analysis only considered peer-reviewed publications in English.

Exclusion criteria included: preprints, letters to editors, conference abstracts, protocols, studies with unclear details of efficacy measurement, qualitative studies, survey reviews, unable to obtain full-text versions for evaluation, and studies focusing only on application program interfaces or internal structures.

### Research screening and data extraction

Literature screening and data extraction were conducted by two researchers, with any disagreements resolved through consultation with a third investigator. All retrieved records were exported to EndNote X9 (Clarivate Analytics), where duplicate publications were identified and removed, first via the automated duplicate detection feature and then through manual verification. Subsequently, the full texts of potentially eligible studies were obtained and assessed based on the established inclusion and exclusion criteria.

Data were extracted using Excel tables, which included the following variables: first author, year of publication, type of study, age range, sample size, country of origin, remote digital health management interventions, duration of intervention, involvement of medical professionals, strategies to ensure subject compliance, and research outcomes.

### Research quality evaluation

The Cochrane Risk of Bias tool (RoB 2.0) was utilized to assess the methodological quality of the included studies (14). Two researchers independently conducted the quality assessment, resolving disagreements through discussion and having a third independent investigator review and confirm the final decision. The evaluation encompassed five domains: randomization process, deviations from intended interventions, missing outcome data, measurement of the outcome, and selection of the reported result. Each domain was categorized as having a low, high, or some concerns risk of bias.

### Statistical analysis

STATA 18.0 software was used for statistical analysis and heterogeneity test of the data. The enumeration data were expressed as relative risk ratio (RR), and the measurement data were expressed as standardized mean difference (SMD) or weighted mean difference (WMD) and 95% confidence interval (CI). A Chi-square test was performed to evaluate heterogeneity among the studies. If *P* < 0.10, *I*^2^ ≥ 50%, it indicated that the heterogeneity among the studies was large, and the random effect model was used for analysis. Otherwise, a fixed-effects model was used. Funnel plot and Egger's test were used to test publication bias, applicable solely to meta-analyses that included a minimum of 10 studies. *P* < 0.05 was considered statistically significant.

For the enumeration data (emergency visits and hospitalizations), we preferentially extracted the proportion of participants with ≥1 event. Trials reporting only person-time rates or mean event counts were excluded from the primary RR meta-analysis unless dichotomous data could be reliably derived. For the primary meta-analysis of each outcome, we extracted data from the longest follow-up time point reported by each trial. To explore temporal patterns, we conducted subgroup analyses stratified by follow-up duration (3, 6, and 12 months), with each study contributing to the relevant time strata without cross-time pooling.

### Evidence quality evaluation

The quality of evidence for primary outcomes was evaluated using GRADEpro. Each outcome received a rating of high, moderate, low, or very low depending on factors such as risk of bias, inconsistency, imprecision, indirectness, and publication bias. These results were summarized in a table of the Supplementary File 2.

## Results

### Research screening

**Figure 1:**
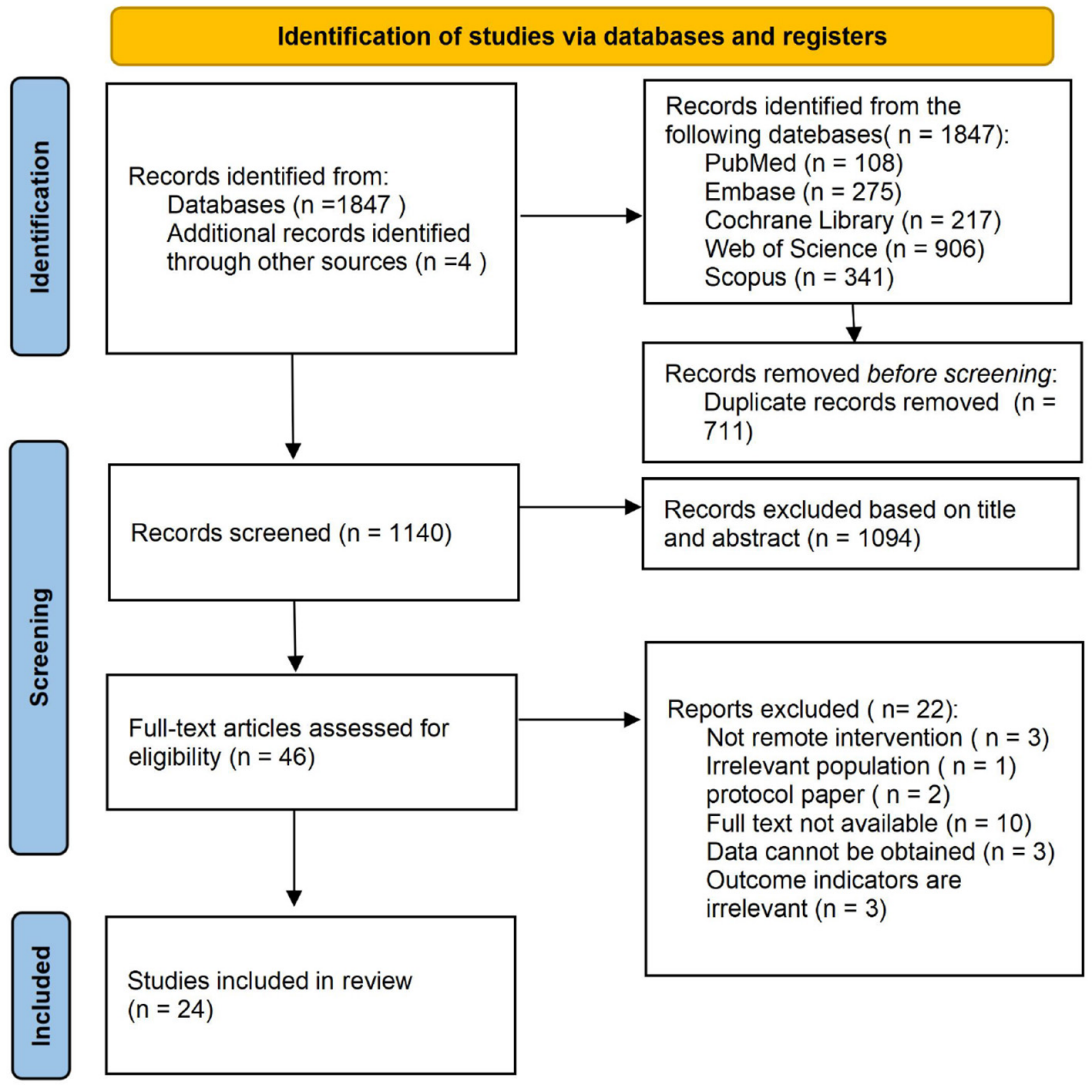
The PRISMA (Preferred Reporting Items for Systematic Reviews and Meta-Analysis) flow diagram.

The PRISMA flow chart (Figure 1) shows the process of literature screening and exclusion criteria. A total of 1,847 records were retrieved from PubMed, Embase, Cochrane Library, Web of Science and Scopus databases, and 4 records were identified from other sources. After removing 711 duplicate records, the remaining 1,136 records were screened for titles and abstracts according to the inclusion and exclusion criteria. Subsequently, 46 full-text literatures were further evaluated. Among them, 22 articles were excluded for the following reasons: non-remote intervention (*n* = 3), data cannot be obtained (*n* = 3), outcome indicators are irrelevant (*n* = 3), irrelevant population (*n* = 1), protocol paper (*n* = 2), and full text not available (*n* = 10). After screening, 24 articles were finally included in this systematic review and meta-analysis.

### Research quality analysis

**Figure 2:**
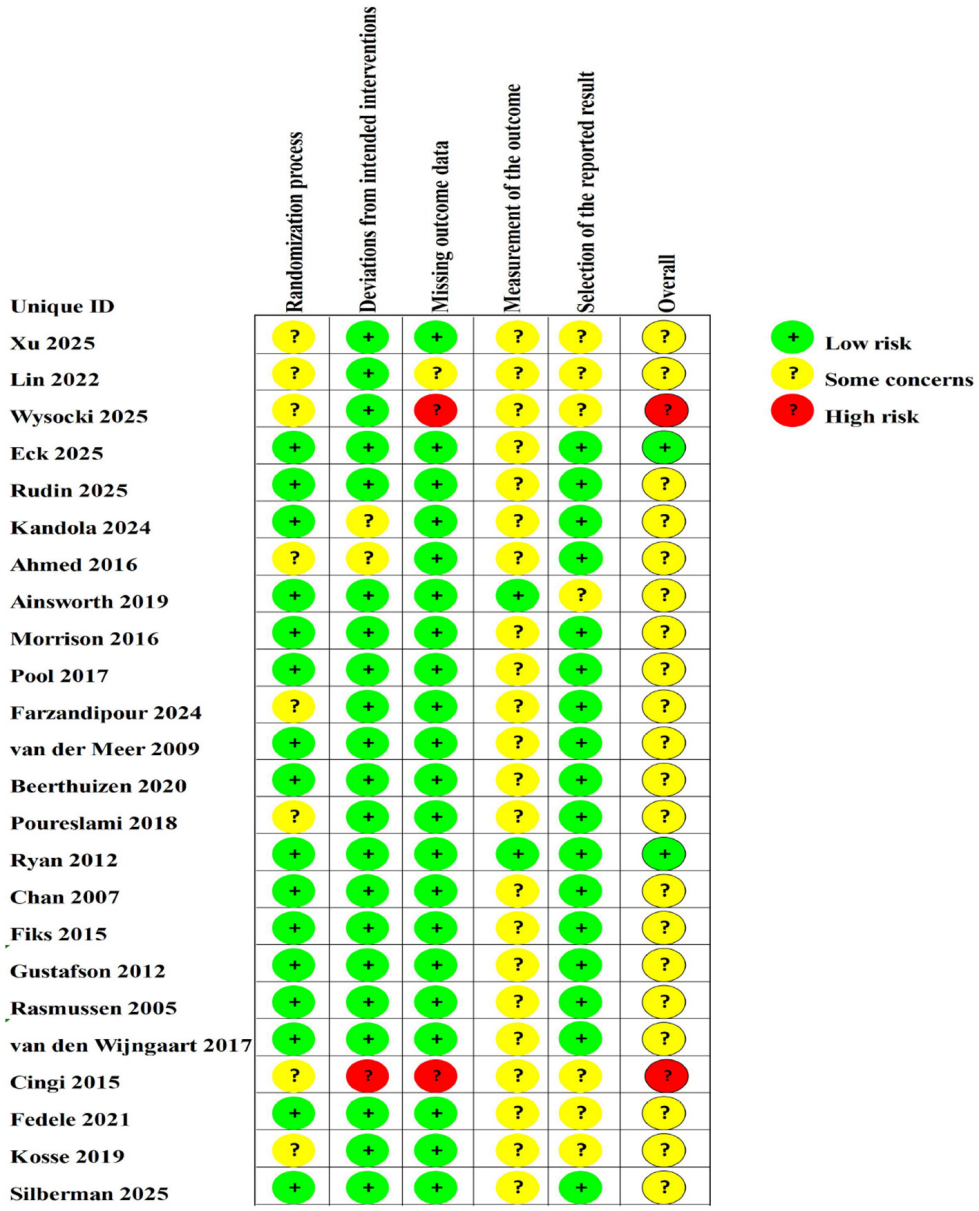
Risk of bias summary of randomized controlled trials.

The bias risk assessment summary is depicted in Figure 2. Among the 24 RCTs, a high risk of bias was observed primarily attributed to the inherent nature of digital health management interventions, rendering blinding of researchers and participants impractical and leading to inevitable risk deviation. All studies employed specific random sequence generation methods, with 9 (37.5%) raising “some concerns,” primarily stemming from unclear allocation concealment. Twenty-one studies exhibited a low risk of deviating from expected interventions and missing outcome data. Sixteen studies demonstrated a low risk in reporting bias. Due to the challenges in implementing blinding due to intervention characteristics, only 2 trials showed a low risk in outcome measurement.

### Characteristics of included studies

A total of 24 studies were published between 2005 and 2025, encompassing 5,155 participants, with sample sizes ranging from 33 (15) to 923 (16). Geographically, 7 studies (15, 17–22) were conducted in the United States, 5 in the UK (23–27), 4 in the Netherlands (28–31), 2 studies in China (16, 32) and Canada (33, 34), and 1 study in Denmark (35), Germany (36), Iran (37), and Turkey (38). In terms of research design, all studies were RCTs, of which 21 literatures (16–25, 27–33, 35–38) reported clinical outcomes, and 3 were pilot study (15, 26, 34). In terms of duration of intervention, 8 studies lasted ≤ 3 months (15, 23–26, 32, 36, 38), 6 studies (21, 27, 30, 33, 35, 37) lasted between 3 and 6 months, and 10 studies (16–20, 22, 28, 29, 31, 34) lasted ≥12 months. In terms of age demographics, 7 studies focused on minors, while the remaining studies included participants aged 18 years and older.

In 11 trials, the control group received standard treatment without the application (21–23, 26, 28–31, 33, 37). In three trials, the control group was provided with either general asthma guidance (18), standardized office-based care (20), or specialist care at an outpatient clinic via mail (35). Five trials involved the control group receiving face-to-face education, paper diary manuals, or paper monitoring (15, 16, 27, 32, 36). Additionally, in two trials, the control group received a routine care manual (25) and a Written Asthma Action Plan (AAP) (34). In four trials, the control group used the same app as the intervention group, but with limited functionality (17, 24, 38) or received feedback on non-asthma-related issues (19). The data extracted from the included studies are summarized in Table 1.

**Table 1:** Detailed characteristics of included studies.

References	Countries	Study design	Study type	Participants, *n*	Age (y), mean (SD)	Intervention and control arm	Intervention duration, *n*	Involvement of health care professionals	Measures to ensure intervention adherence	Outcomes
Silberman et al. (17)	United States	Clinical outcome	RCT^a^	450/449	E^b^: 36.7 (10.4); C^c^: 36.5 (10.6)	E: digital asthma self-management (DASM); C: a modified app(not provide DASM features)	12 months	no	Each notification encouraged the participant to complete a daily entry	ACT^d^
Xu et al. (32)	China	Clinical outcome	RCT	53/50	E: 42.89 (10.82); C: 44.3 (10.25)	E: WeChat Mini Program; C: face-to-face education + diary handbook	3 months	Pharmacists	Auto-reminder messages from the program	ACT; FEV1%/ predicted; AQLQ^e^; Emergency visits; Hospitalization
Lin et al. (16)	China	Clinical outcome	RCT	461/462	E: 44.30 (14.87); C: 44.95 (15.62)	E: Mobile App; C: traditional paper diaries.	12 months	Physicians	App reminders, doctor reminders	ACT; Mini-AQLQ^f^; ${\text{FEV}}_{1}^{\text{g}}$; Emergency visits; Hospitalization
Wysocki et al. (23)	UK	Clinical outcome	RCT	22/23	E: 18 (1.9); C: 18 (1.6)	E: Use of ASTHMAXcel Perception app with PEF feedback; C: Usual care	1.5 months	research assistant	remote intervention sessions	ACT; mini-AQLQ; Emergency visits; Hospitalization
Rudin et al. (18)	United States	Clinical outcome	RCT	211/202	E: 51.9 (15.5); C: 52.6 (15.4)	E: a mobile health app; C: Usual care + general asthma guidance via email.	12 months	study nurse, physician	App email reminder	mini-AQLQ
Kandola et al. (24)	UK	Clinical outcome	RCT	67/85	E: 35.73 (11.48); C: 36.62 (13.23)	E: Full version of the juli app; C: Limited attention-placebo control version of the app	2 months	None specified	Automated daily prompts to open the app; prize draw incentives.	ACT
Eck et al. (36)	Germany	Clinical outcome	RCT	62/46	E: 43.9 (15.3); C: 50.1 (13.8)	E: Access to eAEP (online asthma education program); C: Usual Care, including referral to a face-to-face AEP (fAEP)	2 weeks	general practitioner	Phone/Mail Questionnaire Reminder	ACT; Emergency visits; Hospitalization
Ahmed et al. (33)	Canada	Clinical outcome	RCT	47/51	/	E: Access to MAP (web portal) + Nurse Case Manager support; C: Usual care	6 months	Nurse Case Manager	Automated alerts for non-login; Escalation to nurse after 48 h of inaction.	mini-AQLQ
Ainsworth et al. (25)	UK	Clinical outcome	RCT	44/44	E: 57.0 (14.2); C: 56.3 (16.2)	E: usual care with MBM and asthma UK booklet; C: usual care with asthma UK booklet	3 months	no	Automated reminders for non-use; One tech support call/email if no initial login.	mini-AQLQ; ACQ; FEV_1_; FEV_1_/FVC^h^; FEV1% predicted
Morrison et al. (26)	UK	Pilot study	RCT	25/26	E: 44.6 (17); C: 46.4 (14)	E: website access; C: usual care.	3 months	no	email reminders	ACQ^i^; mini-AQLQ; FEV_1_; FEV_1_/FVC; FEV_1_% predicted; Emergency visits
Pool et al. (19)	United States	Clinical outcome	RCT	203/204	E: 47.6 (9.1); C: 31.0 (7.6)	E: Online tool; C: Active control Online tool with questions/feedback on preventive services unrelated to asthma.	12 months	general internist;specialist	email reminders	ACT; Emergency visits
Farzandipour et al. (37)	Iran	Clinical outcome	RCT	30/30	E: 39.03 (15.51); C: 40.4 (15.74)	E: Usual care + RespRight smartphone app; C: usual care	6 months	Physicians, the clinic secretary	Not described	ACT
van der Meer et al. (28)	Netherlands	Clinical outcome	RCT	101/99	E: 36 (19–50); C: 37 (18–50)	E: Internet-based self-management; C: usual care	12 months	Specialized asthma nurse	Web communication	AQLQ; ACQ; FEV_1_
Beerthuizen et al. (29)	Netherlands	Clinical outcome	RCT	33/29	E: 46.7 (2.3); C: 44 (2.4)	E: Internet-based self-management support (PatientCoach) + Usual care; C: Usual care.	12 months	No	Not described	ACQ6^j^; AQLQ
Poureslami et al. (34)	Canada	Pilot study	RCT	52/54	E: 50.6 (15.3); C: 52.8 (15)	E: The digitalized asthma action plan (eAAP); C: Written Asthma Action Plan (AAP)	12 months	Respiratory educators	telephone	FEV_1_
Ryan et al. (27)	UK	Clinical outcome	RCT	145/143	E: 46.6 (18); C: 51.5 (17.7)	E: Mobile phone-based monitoring (t+ Asthma app); C: Paper-based monitoring	6 months	Practice asthma nurses	Technical support calls.	ACQ; mini-AQLQ; Emergency visits; Hospitalizations
Fedele et al. (15)	United States	Pilot study	RCT	17/16	E: 13.42 (1.23); C: 12.94 (1.06)	E: AIM2ACT mHealth intervention; C: paper-based versions of the surveys	2 months	no	Not described	ACT; FEV_1_; PAQLQ^k^
Kosse et al. (30)	Netherlands	Clinical outcome	RCT	87/147	E: 15 (2); C: 15.2 (1.9)	E: ADAPT mHealth E; C: Usual care	6 months	Community Pharmacists	Medication reminder alarms, adherence questionnaire monitoring, pharmacist Chat and Training Features.	CARAT^l^; PAQLQ
Cingi et al. (38)	Turkey	Clinical outcome	RCT	60/29	E: 32.0 (3.7); C: 34.5 (8.2)	E:Use a mobile app for remote monitoring; C:Use of a limited app	3 months	Physicians	App-based medication reminders	ACT; Emergency visits
Chan et al. (20)	United States	Clinical outcome	RCT	60/60	E: 10.2 (3.1); C: 9 (3)	E: Internet-based home monitoring & education; C:Standardized office-based care.	12 months	The pediatric clinical pharmacist case manager; the study pediatrician; 4 assigned nurse case managers	Regular contact (phone/email); reminders for diary and video submission.	PAQLQ; FEV_1_% predicted; Emergency visits; Hospitalization
Fiks et al. (21)	United States	Clinical outcome	RCT	30/30	E: 8.3 (1.9); C: 8.2 (1.9)	E: MyAsthma portal; C: Usual care	6 months	Clinicians; primary care providers	Email reminders for monthly surveys; integration with EHR	Emergency visits; Hospitalization
Rasmussen et al. (35)	Denmark	Clinical outcome	RCT	85/80	E: 28.38 (5.33); C: 30.33(5.17)	E:Internet-based management tool; C: Specialist care outpatient clinic	6 months	Physicians (specialists)	Usage data extracted from the tool	Emergency visits
van den Wijngaart et al. (31)	Netherlands	Clinical outcome	RCT	105/105	E: 11.3 (2.9); C: 11.3 (2.7)	E: Virtual Asthma Clinic (VAC); C: Usual care	16 months	Asthma team; treating physicians	Automated reminders after 1 and 2 weeks for missing questionnaires	ACT; FEV_1_% predicted; Emergency visits; Hospitalization
Gustafson et al. (22)	United States	Clinical outcome	RCT	148/153	E: 7.65 (2.61); C: 8.18 (2.45)	E:Access to the CHESS (Comprehensive Health Enhancement Support System) eHealth program; C: Usual care	12 months	University-based asthma nurse case managers	Monthly reminder calls from the case manager	ACQ

^a^RCT, randomized controlled trial.

^b^E, experimental group.

^c^C, control group.

^d^ACT, Asthma Control Test.

^e^AQLQ, Asthma Quality of Life Questionnaire.

^f^mini-AQLQ, Mini Asthma Quality of Life Questionnaire.

^g^FEV_1_, Forced expiratory volume in 1 s.

^h^FVC, forced vital capacity.

^i^ACQ, Asthma Control Questionnaire.

^j^ACQ6, Asthma Control Questionnaire 6-item version.

^k^PAQLQ, Pediatric Asthma Quality of Life Questionnaire.

^l^CARAT, Control of Allergic Rhinitis and Asthma Test.

### Involvement of healthcare professionals and strategies to ensure compliance through digital health tool management interventions

A total of 18 trials (16, 18–23, 27, 28, 30–38) included data on the use of digital health tools by health care professionals, while the remaining six trials (15, 17, 24–26, 29) did not provide specific information on the use of digital health tools by health care professionals (Table 1). The roles of health care professionals in each trial are different. In most cases, they are mainly responsible for providing asthma-related education and intervention treatment for patients (19–22, 27, 28, 30–35, 37), installing digital health equipment and guiding patient use (20, 36, 37), follow-up data collection (27, 33, 34) and evaluation of patient data (16, 20, 22, 23, 31, 38). Two trials described specific strategies for improving adherence to digital health tool management interventions, including monitoring progress (20, 33), sending regular email reminders, and regular telephone follow-up (20).

### Features of digital medical tools

A total of 24 different digital health tools were used in various studies. These tools are deployed on a variety of digital platforms, including one WeChat applet (32), 12 apps (15–18, 23, 24, 27, 29, 30, 34, 37, 38), eight websites (19–21, 26, 28, 31, 33, 36), and three tools with unclear platform details (22, 25, 35). The function of each test platform is significantly different. Digital interventions are mostly focused on topics such as clinical outcome measurement, self-monitoring of disease, or drug compliance. In terms of self-management, most digital health platforms have functions such as self-monitoring, medication reminders, health information push, evaluation feedback, medical service access, and social support. Some platforms can track various physical activities and transmit relevant data to the server for the therapist to consult, so as to formulate more targeted treatment plans. The characteristics of digital health tools are shown in Table 2.

**Table 2:** Digital health tools characteristics.

References	Digital health tools name	General objective	Specific objectives	Devices	Main features of the digital health tools
Silberman et al. (17)	MyDataHelps	Self-management	Asthma symptom control in relation to self-management behaviors	Smartphone (App); sleep monitor (Apple Inc.)	Tailored notifications; symptom logging; and evidence-based education.
Xu et al. (32)	WeChat Mini Program “Asthma Self-management”	Self-management	Improve asthma self-management; control; and quality of life.	Smartphone with WeChat app	Asthma diary & ACT; Educational articles/videos; Auto-reminders for medication/diary; Online consultation portal; Inhaler technique video upload/feedback.
Lin et al. (16)	Mobile Asthma Evaluation and Management System	Self-management	Improve adherence and control of asthma.	Smartphone (App)	Electronic diary; medication/visit reminders; educational info; real-time feedback based on PEF/ACT scores; physician monitoring platform.
Wysocki et al. (23)	ASTHMAXcel Perception	Self-management	Improve asthma control and perception of airflow limitation.	iOS/Android smartphone/tablet; Microlife PF100 electronic peak flow meter.	PEF guess vs. actual with immediate feedback; Gamified interface; Educational videos/chapters; Goal-setting; Medication/trigger reminders; Leaderboard.
Rudin et al. (18)	Not stated	Self-management	Increase patient awareness of symptoms; facilitate clinical support when needed; improve asthma-related quality of life.	Smartphone (App)	EHR-integrated; weekly 5-item symptom questionnaire; symptom graphs; educational materials; callback request for worsening symptoms; available in English and Spanish.
Kandola et al. (24)	juli	Self-management	Improve the self-management ability of patients and the control of asthma	Smartphone (iOS/Android)	Symptom tracking, medication reminders; trigger ID (weather/pollen/air quality); data visualization, behavioral activation; positive affect journaling.
Eck et al. (36)	eAEP	Self-management	Improve asthma self-management and knowledge.	PC; laptop; or smartphone (via website).	Structured online education (asthma basics, medication, self-management, exacerbations), GP-supervised follow-up.
Ahmed et al. (33)	MyAsthma Portal (MAP)	Self-management	Improve asthma-related QoL and control via monitoring and feedback.	Computer/Web	Self-monitoring (symptoms, activity, meds); Personalized feedback; Educational links; Communication with nurse.
Ainsworth et al. (25)	My Breathing Matters (MBM)	Self-management	Improve asthma specific quality of life	Computer/Web	Tailored advice; Pharmacological (meds, action plans) and non-pharmacological (breathing, stress) modules; Self-monitoring.
Morrison et al. (26)	Living Well with Asthma	Self-management	Support self-management	desktop/laptop	Support optimal medication management; promote use of action plans; encourage attendance at asthma reviews and increase physical activity.
Pool et al. (19)	Not stated	Self-management	Improve asthma control	desktop/laptop	Remind patients to ask providers for guideline-based treatments and to perform specific self-care behaviors (e.g., proper inhaler technique).
Farzandipour et al. (37)	RespRight	Self-management	Improve asthma control and quality of life via self-management.	Android Smartphone	Symptom & PEF logging; educational content; medication reminders; communication with physician; self-assessment tools.
van der Meer et al. (28)	Not stated	Self-management	Support patient self-management	Home computer/device with internet; PIKo-1 electronic spirometer.	Weekly ACQ monitoring with automated treatment advice; Online education repository; Remote communication with nurse; Daily symptom/FEV1 tracking (optional).
Beerthuizen et al. (29)	PatientCoach	Self-management	Sustain self-management post-rehabilitation.	Computer/Web/Mobile	Personal action plan; Daily symptom & lung function (FEV_1_) monitoring; Activity tracker (Fitbit); Personal health goals; Educational material.
Poureslami et al. (34)	WeITel mHealth platform	Self-management	Improve self-management and reduce exacerbations.	Mobile Phone + Web	Trigger the participant to assess their asthma control each week; and if they experienced a worsening of asthma symptoms; be linked to an electronic version of their individual AAP.
Ryan et al. (27)	t+ Asthma	Self-management	Improve asthma self-management and control.	Compatible mobile phone handset; Piko meter for PEF.	Twice-daily data recording/transmission; Traffic light feedback system (PEF zones); Automated prompts for action plan; Triggers nurse contact; Secure web access to data.
Fedele et al. (15)	AIM2ACT	Self-management	Increase helpful caregiver support as early adolescents engage in asthma self-management behaviors	Smartphone	Ecological Momentary Assessment (EMA) twice daily; Graphical feedback on asthma impairment and management; Goal setting and interactive behavioral contracting between adolescent and caregiver; Animated skills-training videos (tailored for adolescents and caregivers); Problem-solving communication prompts.
Kosse et al. (30)	ADAPT	Self-management	Support self-management and improving inhaled corticosteroid adherence in adolescents with asthma	smartphone/Desktop computer	Weekly Control of Allergic Rhinitis and Asthma Test (CARAT); Short educational and motivational movies on asthma related to pics; Medication reminder alarm to prevent forgetting; Peer chat function; Pharmacist chat function; Two questions once every 2 weeks to monitor non-adherence.
Cingi et al. (38)	POPET (Physician on Call Patient Engagement Trial)	Self-management	Improve medication compliance; quality of life; and disease control; reduce hospital visits.	Smartphone	Patient-physician messaging; Health status tracking (7-point scale); Medication diary & reminders; Integrated surveys; Physician dashboard for patient management.
Chan et al. (20)	Customized website	Self-management	Improve therapeutic and disease control outcomes; adherence.	Home computer; internet connection; digital video camera	Store-and-forward telemedicine; secure email; digital video uploads; interactive asthma education.
Fiks et al. (21)	MyAsthma (EHR-linked patient portal)	Self-management	Facilitate shared decision-making and improve asthma outcomes.	Computer with internet	EHR-integrated portal; monthly surveys; goal/concern tracking; decision support; educational videos; care plan access.
Rasmussen et al. (35)	Internet-based asthma management tool	Self-management	Improve asthma control.	Computer/Internet or push-button telephone	Electronic diary; automated action plan with 3-color system; decision support system for physician.
van den Wijngaart et al. (31)	Virtual Asthma Clinic (VAC)	Self-management	Reduce outpatient visits by 50%; maintain/improve asthma control.	Computer with internet	Web-based portal, monthly (C-)ACT, automated feedback; educational content; communication module.
Gustafson et al. (22)	CHESS	Self-management	Improve pediatric asthma control and medication adherence.	Computer with internet	“Asthma Coach” for check-ins (symptoms, adherence) with tailored feedback and links to relevant content; medication adherence coach; trigger action plans; journaling.

### Effects of interventions on the health outcomes of patients with asthma

#### Asthma control outcomes

##### Asthma Control Test (ACT)

**Figure 3:**
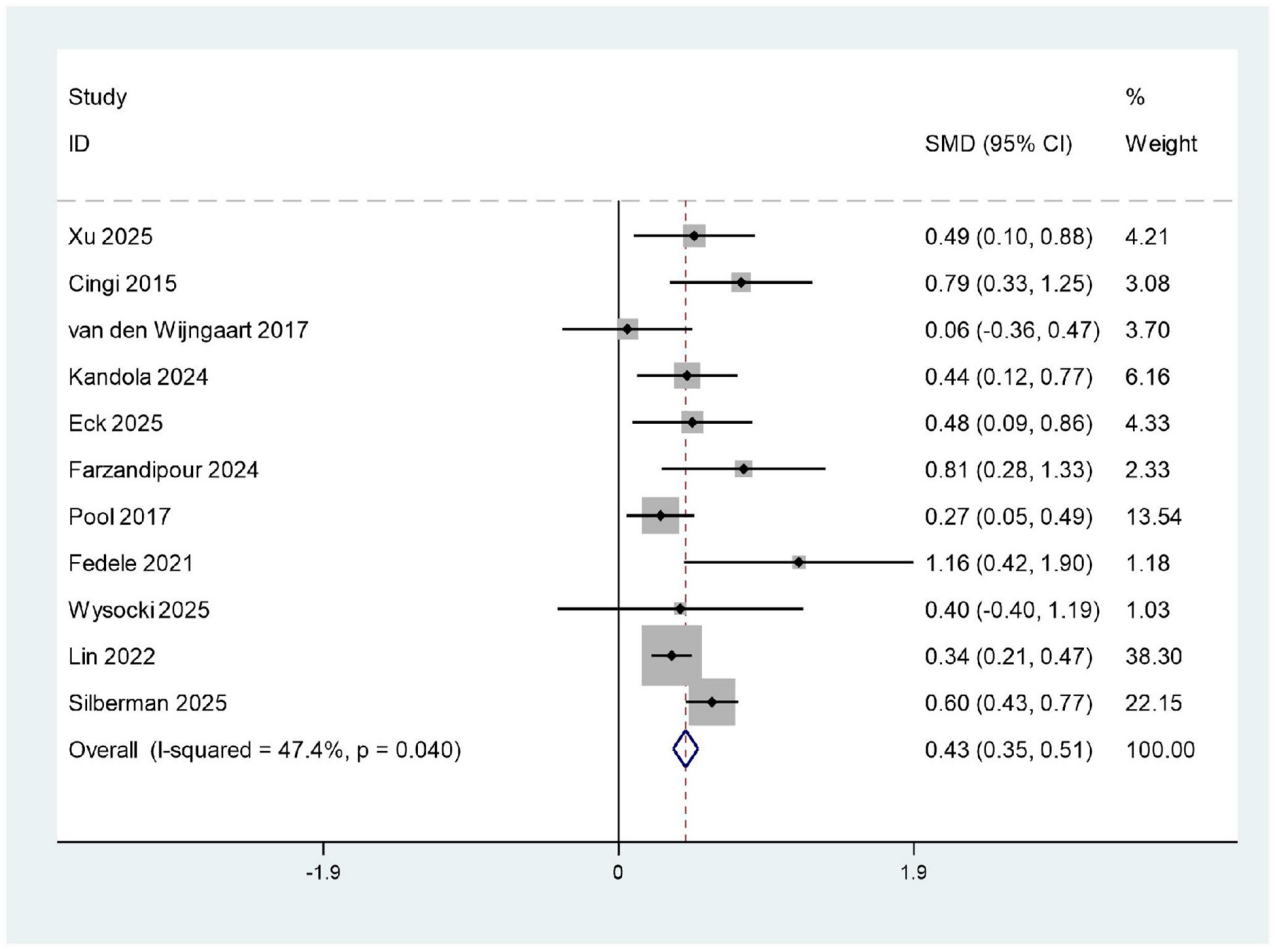
Meta-analysis results and forest plots of digital health interventions for asthma control assessed by ACT.

A total of 11 studies were included in this meta-analysis. The fixed effect model meta-analysis showed that digital health interventions had statistically significant effects [SMD = 0.43, 95% CI: 0.35 to 0.51, *P* < 0.001; Figure 3 (15–17, 19, 23, 24, 31, 32, 36–38)], and there was moderate heterogeneity between studies (*I*^2^ = 47.4%). Sensitivity analysis showed that the results remained robust after excluding any study item by item. Funnel plot (Supplementary File 2) and Egger's test showed no significant publication bias (*P* = 0.271).

**Figure 4:**
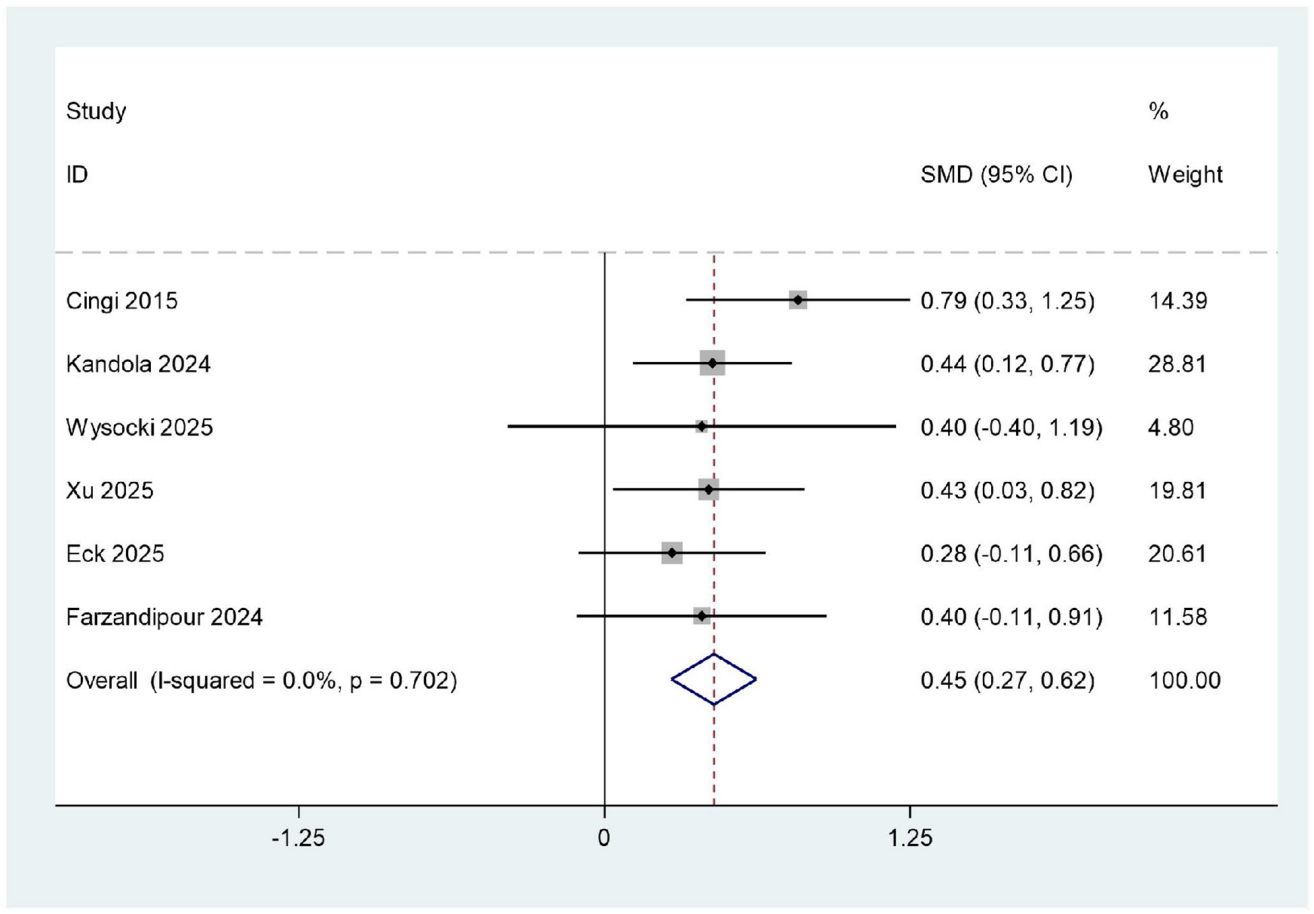
Meta-analysis results and forest plots of digital health interventions for asthma control assessed by ACT at 3 months.

**Figure 5:**
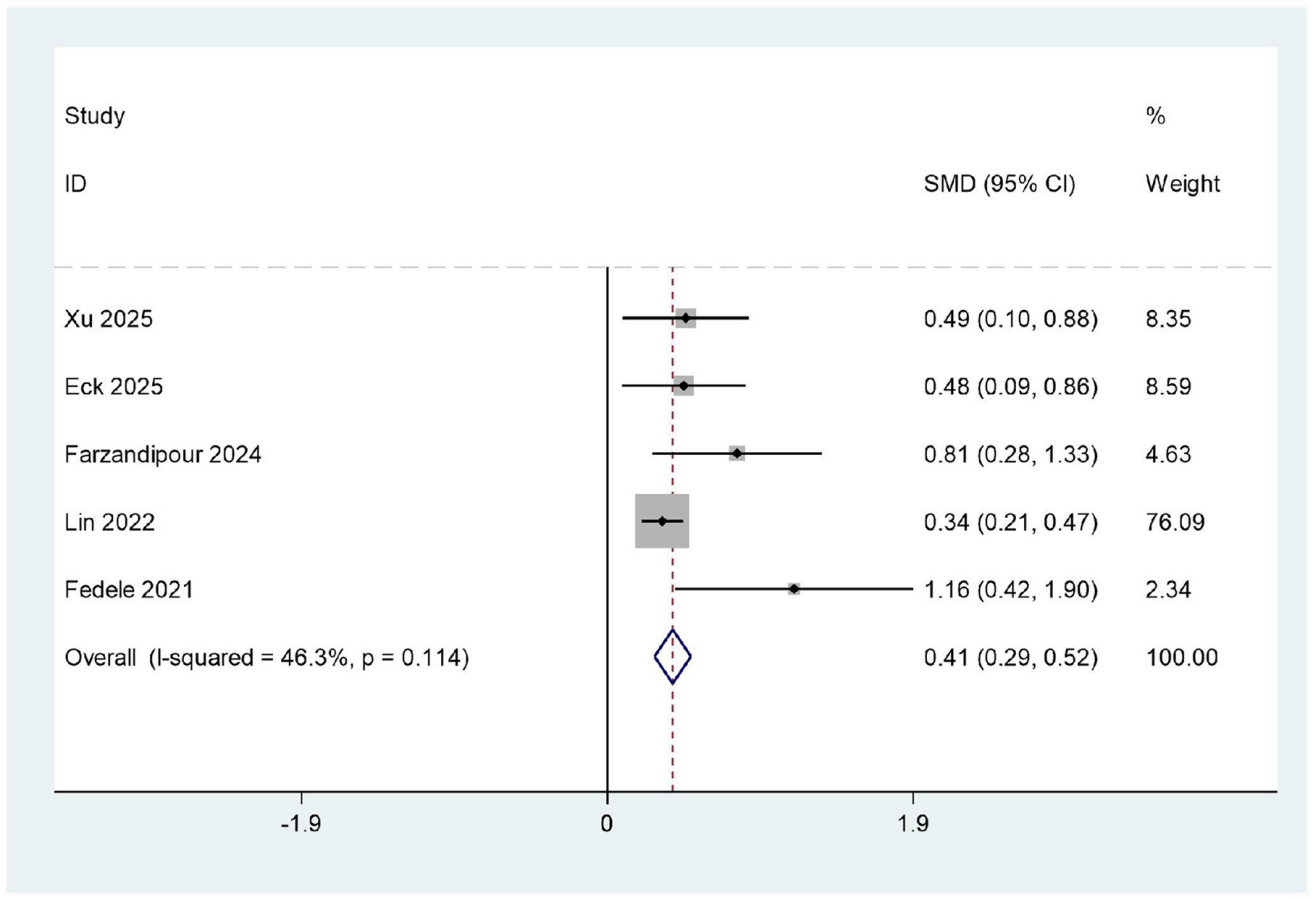
Meta-analysis results and forest plots of asthma control assessed by ACT at 6 months of digital health intervention.

**Figure 6:**
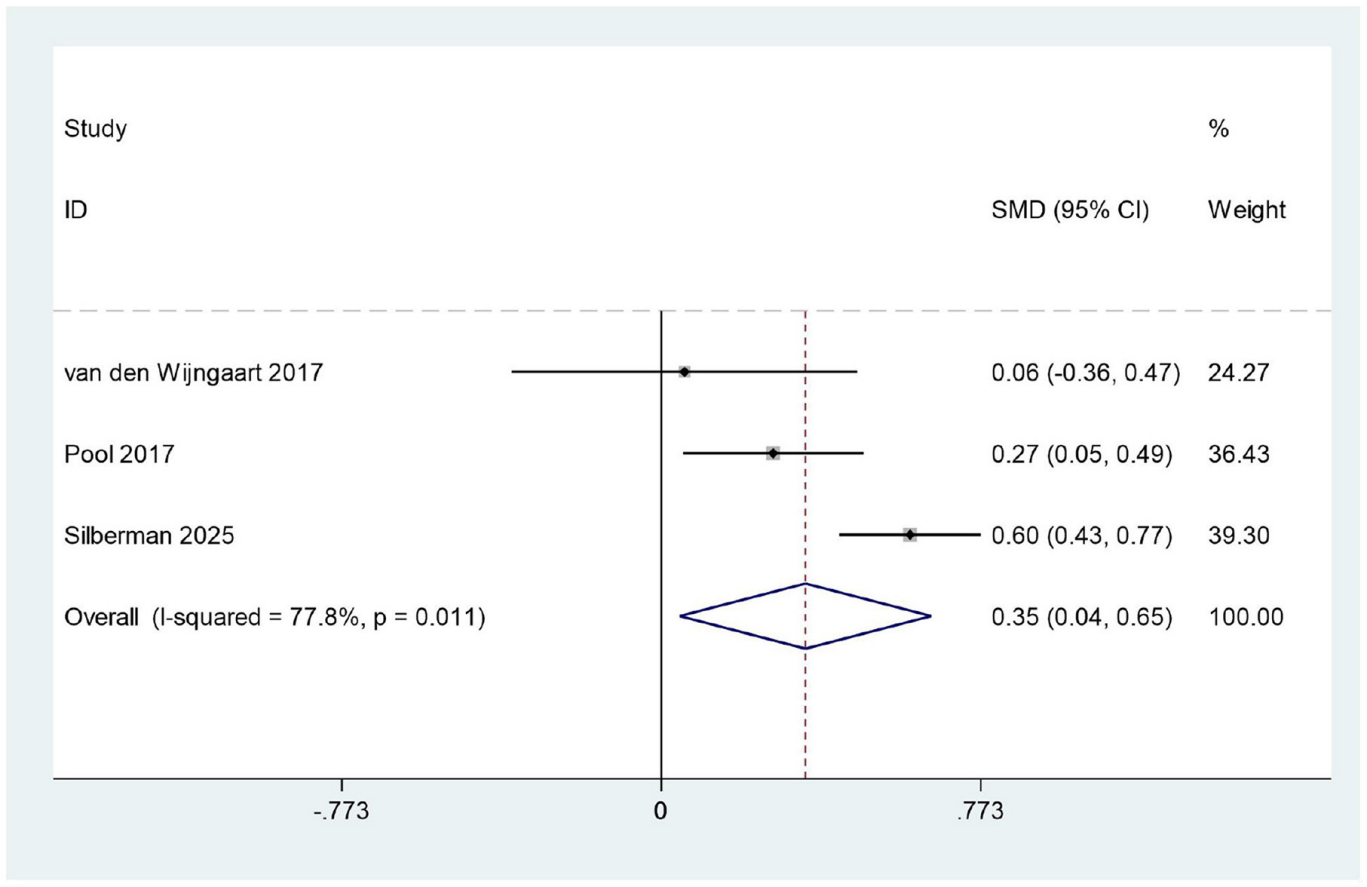
Meta-analysis results and forest plots of asthma control assessed by ACT at 12 months of digital health intervention. Weights are from random effects analysis.

In order to explore heterogeneity, subgroup analysis was performed according to follow-up time. At the 3-month follow-up point (6 studies), the effect was significant and the homogeneity was high [SMD = 0.45, 95% CI: 0.27 to 0.62, *P* < 0.001; *I*^2^ = 0.0%; Figure 4 (23, 24, 32, 36–38)]. At the 6-month follow-up point (5 studies), the effect was still significant [SMD = 0.41, 95% CI: 0.29 to 0.52, *P* < 0.001; Figure 5 (15, 16, 32, 36, 37)], there was moderate heterogeneity (*I*^2^ = 46.3%). At the 12-month follow-up point (3 studies), the results of the random effect model showed that the effect was still significant, but the effect size is slightly lower compared to the 3-month and 6-month effects [SMD = 0.35, 95% CI: 0.04 to 0.65, *P* = 0.025; Figure 6 (17, 19, 31)], and there was a high degree of heterogeneity (*I*^2^ = 77.8 %), suggesting that the long-term effect may be weakened and vary greatly in different studies.

##### Asthma Control Questionnaire (ACQ)

**Figure 7:**
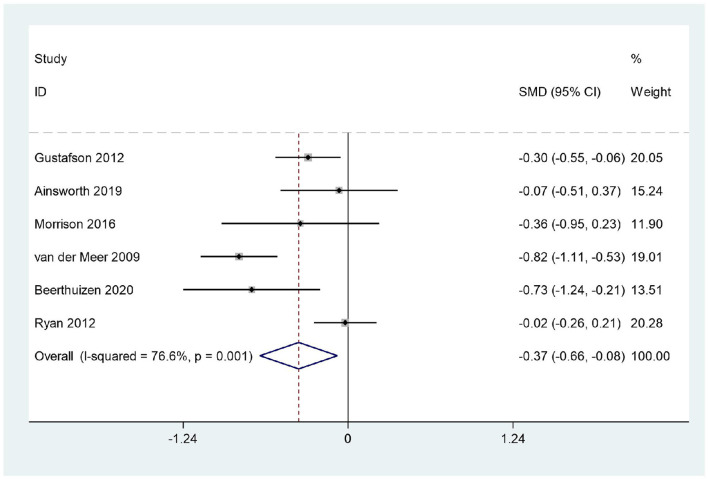
Meta-analysis results and forest plots of digital health interventions for asthma control assessed by ACQ. Weights are from random effects analysis.

A total of 6 studies were included. The combined analysis using a random effect model showed a significant correlation between digital health intervention and ACQ scores of asthma patients [SMD = −0.37, 95% CI: −0.66 to −0.08, *P* = 0.013; Figure 7 (22, 25–29)], suggesting that ACQ scores of asthma patients in the digital health intervention group were significantly lower than those in the control group (the lower the ACQ score, the better the asthma control). The results of sensitivity analysis showed that the exclusion of any study did not significantly change the combined effect size, and the results were robust.

**Figure 8:**
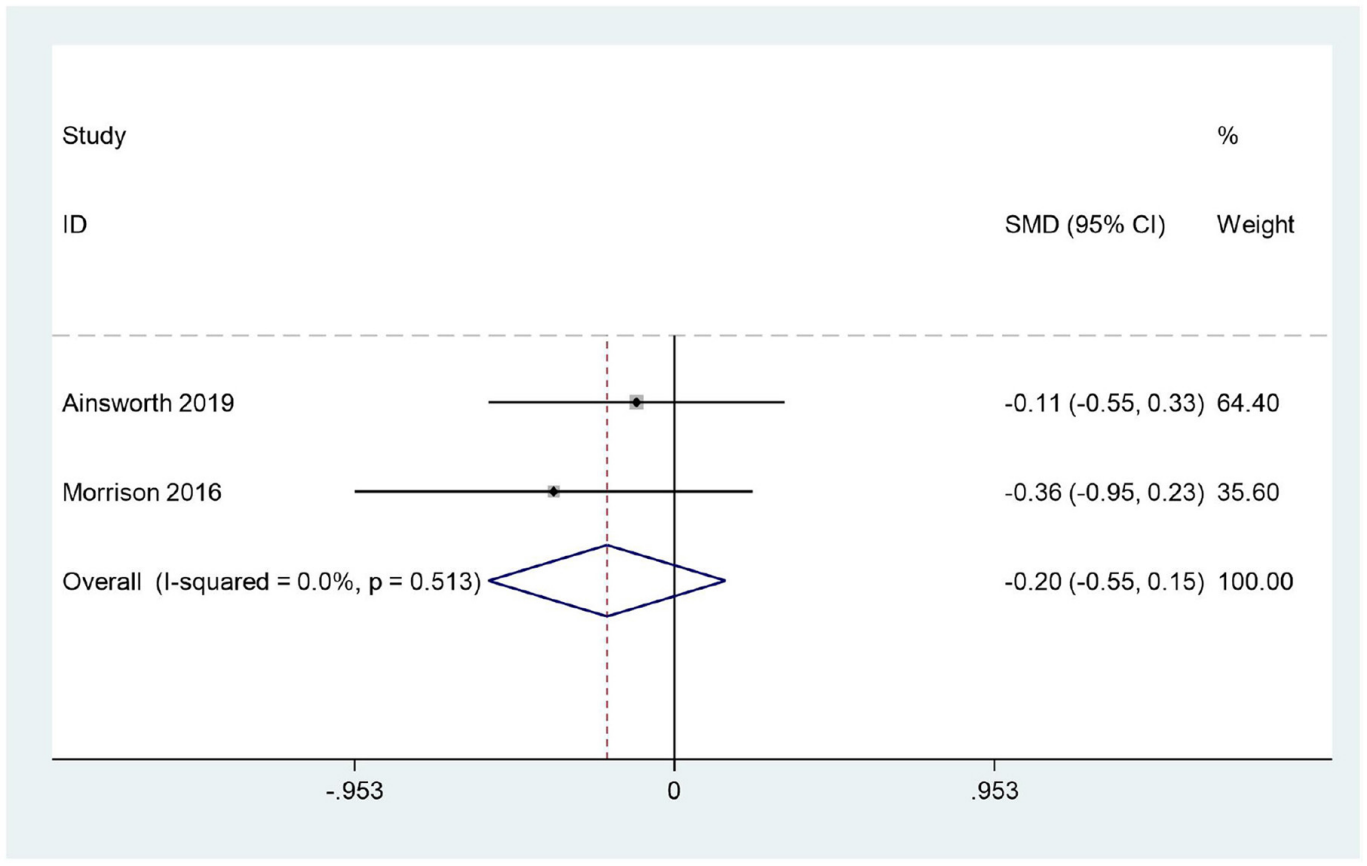
Meta-analysis results and forest plots of digital health interventions on asthma control assessed by ACQ at 3 months.

**Figure 9:**
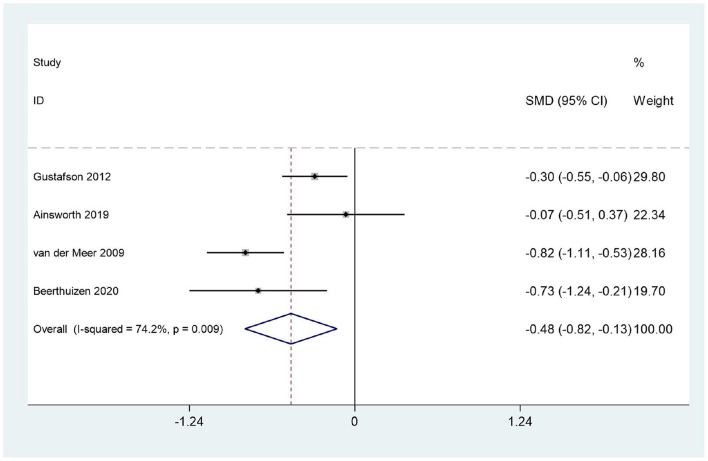
Meta-analysis results and forest plots of digital health interventions for asthma control assessed by ACQ at 12 months. Weights are from random effects analysis.

The subgroup analysis showed that two studies were included in the 3-month follow-up subgroup. The combined analysis showed that the effect of digital health intervention was not statistically significant [SMD = −0.20, 95% CI: −0.56 to 0.15, *P* = 0.265; *I*^2^ = 0.0%; Figure 8 (25, 26)]. A total of 4 studies were included in the 12-month follow-up. The combined analysis showed that digital health intervention had a significant effect [SMD = −0.48, 95% CI: −0.82 to −0.13, *P* = 0.007; Figure 9 (22, 25, 28, 29)], suggesting that long-term (12 months) intervention can significantly improve the ACQ score of asthma patients. Heterogeneity test showed that there was significant moderate heterogeneity within the subgroup (*I*^2^ = 74.2%). The results of subgroup sensitivity analysis showed that the overall positive results of digital health intervention asthma had good robustness. No subgroup analysis was performed due to the insufficient number of 6-month follow-up studies.

#### Quality of life outcomes

##### Asthma Quality of Life Questionnaire (AQLQ)

**Figure 10:**
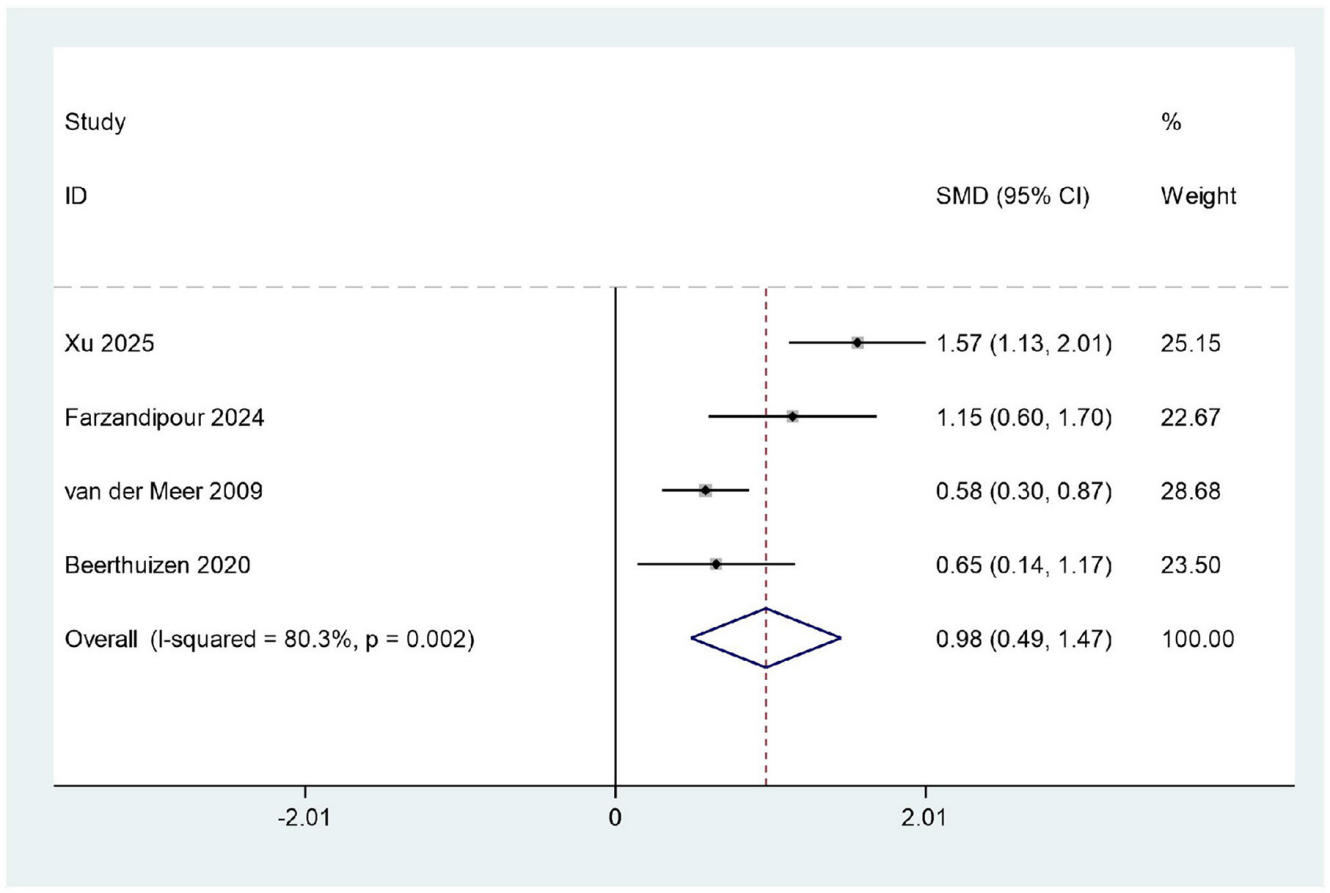
Meta-analysis results and forest map of digital health intervention on asthma quality of life assessed by AQLQ. Weights are from random effects analysis.

**Figure 11:**
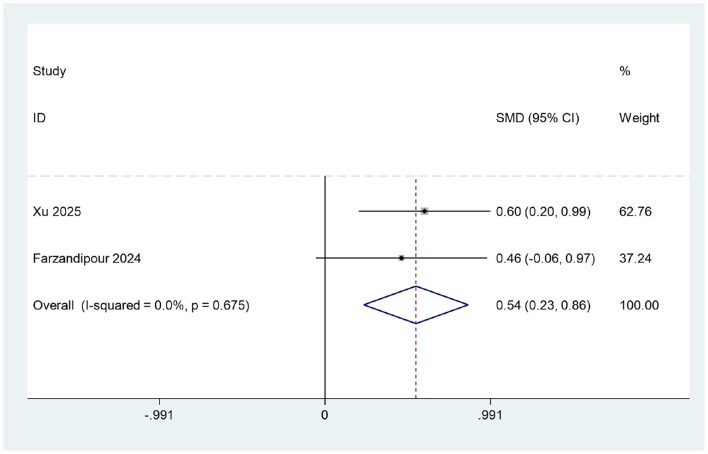
Meta-analysis results and forest map of digital health intervention on asthma quality of life assessed by AQLQ at 3 months.

**Figure 12:**
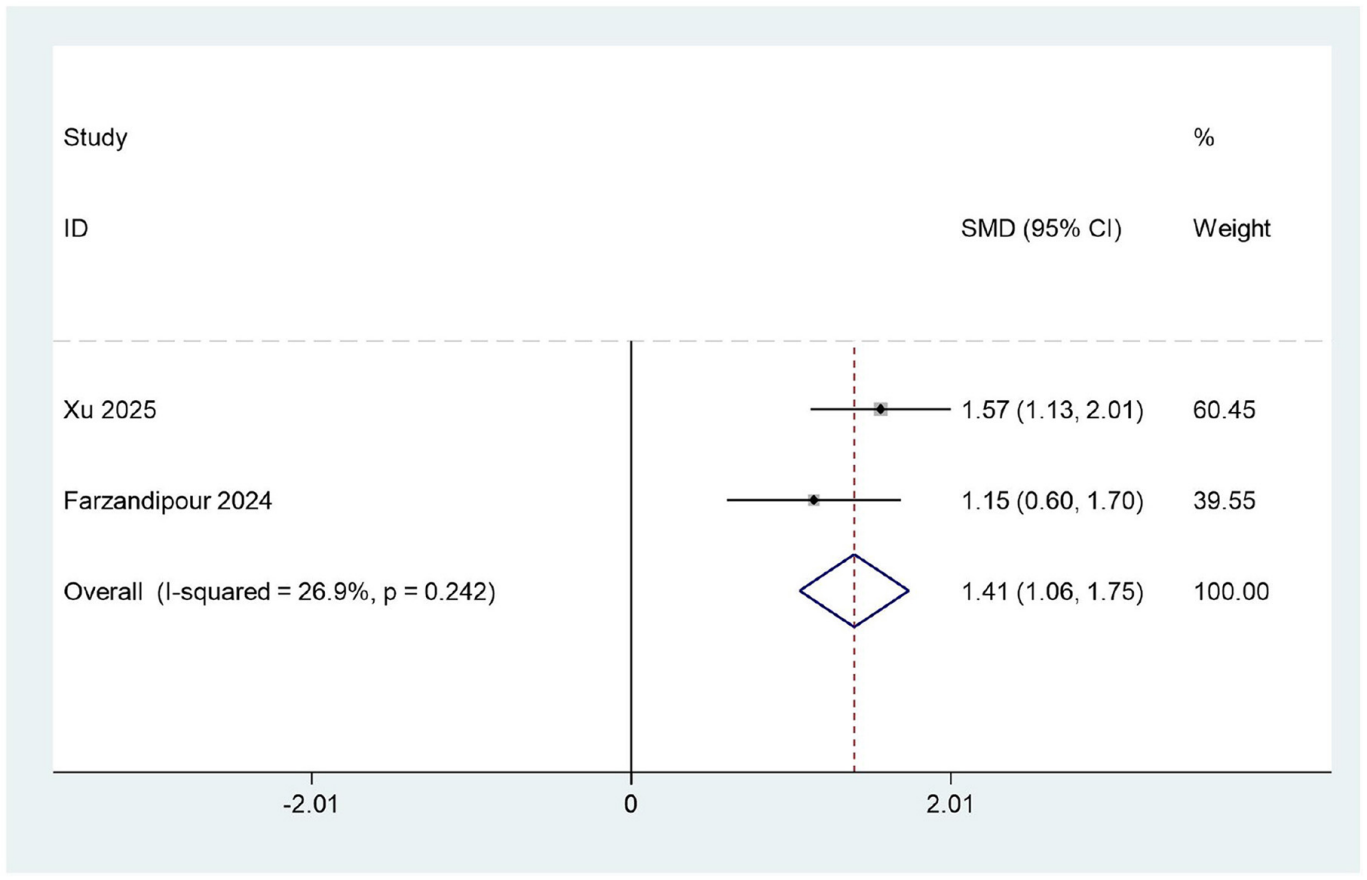
Meta-analysis results and forest map of digital health intervention on the quality of life of asthma assessed by AQLQ at 6 months.

**Figure 13:**
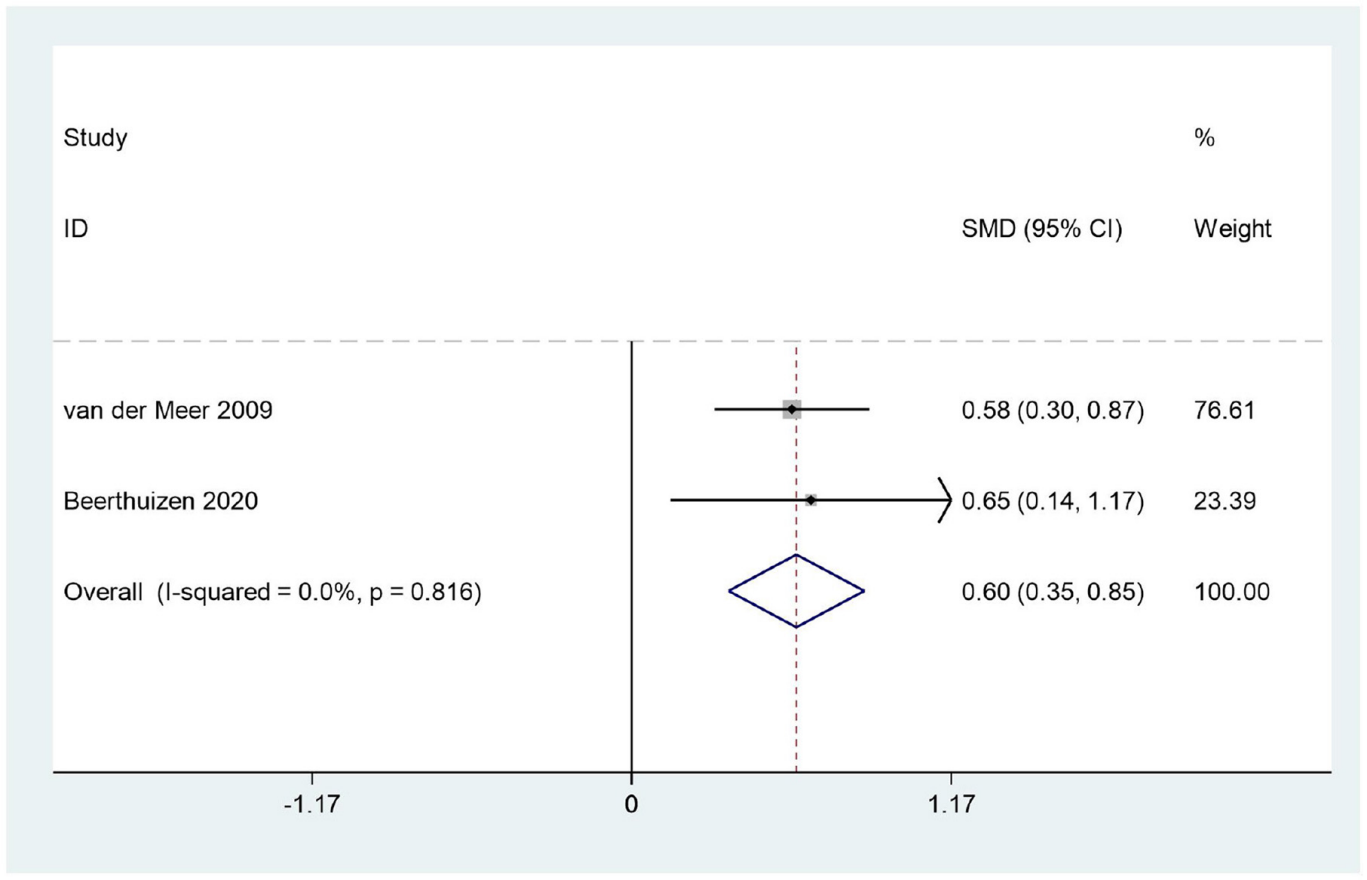
Meta-analysis results and forest plot of digital health intervention on asthma quality of life assessed by AQLQ at 12 months.

The random effect model combined analysis of 4 studies showed that digital health intervention could significantly improve the quality of life of patients, and the effect was large [SMD = 0.98, 95% CI: 0.49 to 1.47, *P* < 0.001; Figure 10 (28, 29, 32, 37)], but there was a high degree of heterogeneity between studies (*I*^2^ = 80.3%). The sensitivity analysis showed that the effect size decreased to 0.60 after excluding the study of Xu et al. (32), indicating that the study was one of the main sources of heterogeneity. Subgroup analysis showed that the effect size was moderate and the heterogeneity was low at 3-month follow-up [SMD = 0.55, 95% CI: 0.23 to 0.86, *P* = 0.001; *I*^2^ = 0.0%; Figure 11 (32, 37)]. At 6 months follow-up, the effect size was the most prominent and the heterogeneity was low [SMD = 1.41, 95% CI: 1.06 to 1.75, *P* < 0.001; *I*^2^ = 26.9%; Figure 12 (32, 37)]. At the 12-month follow-up, the effect size was slightly reduced and the heterogeneity was low [SMD = 0.60, 95% CI: 0.35 to 0.85, *P* < 0.001; *I*^2^ = 0.0%; Figure 13 (28, 29)].

##### Mini Asthma Quality of Life Questionnaire (mini-AQLQ)

**Figure 14:**
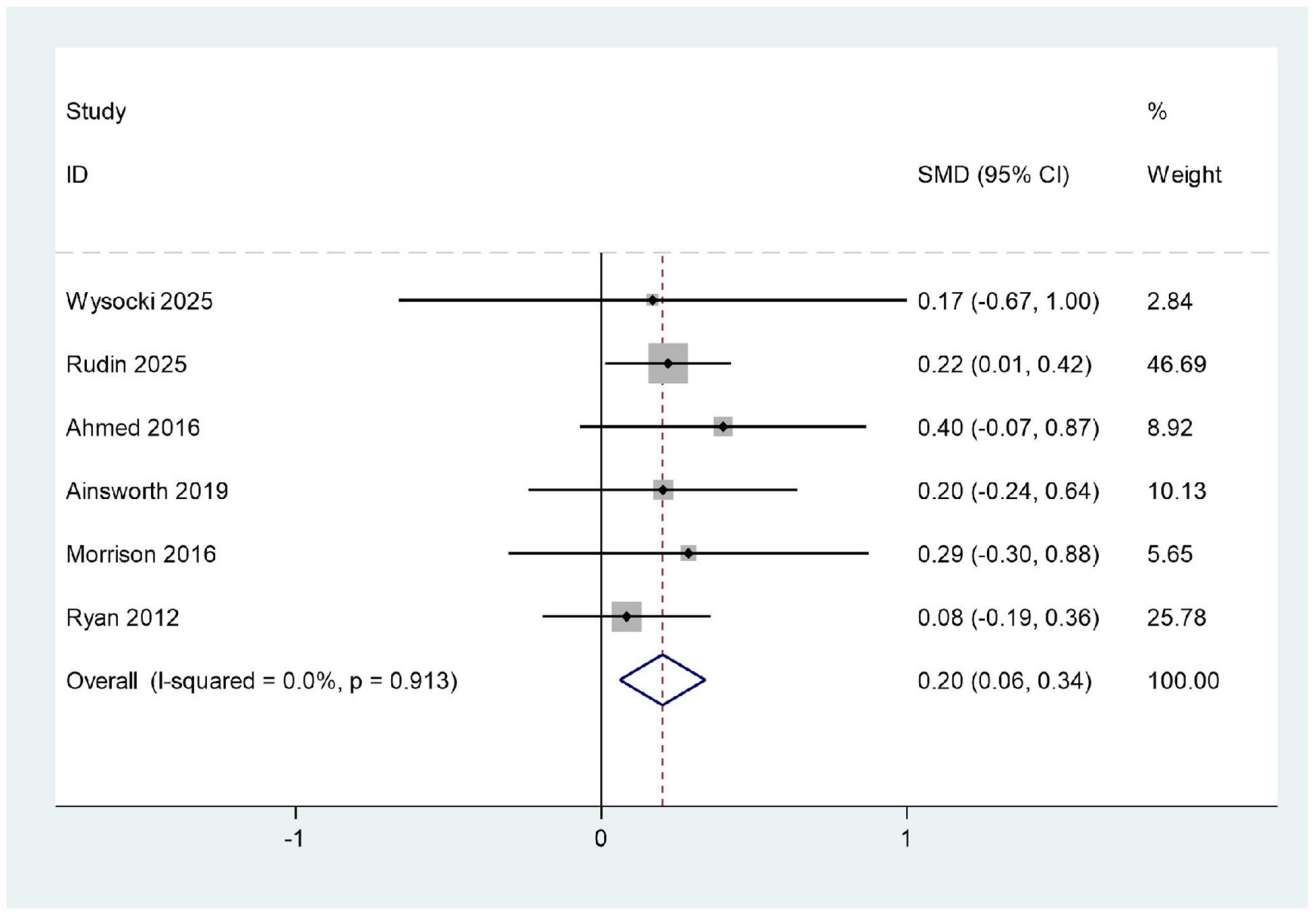
Meta-analysis results and forest map of digital health intervention on the quality of life of asthma assessed by mini-AQLQ.

**Figure 15:**
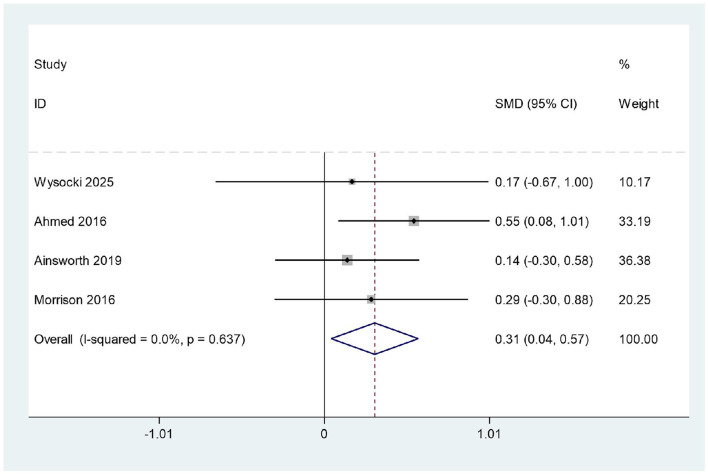
Meta-analysis results and forest plot of digital health intervention on asthma control assessed by mini-AQLQ at 3 months.

**Figure 16:**
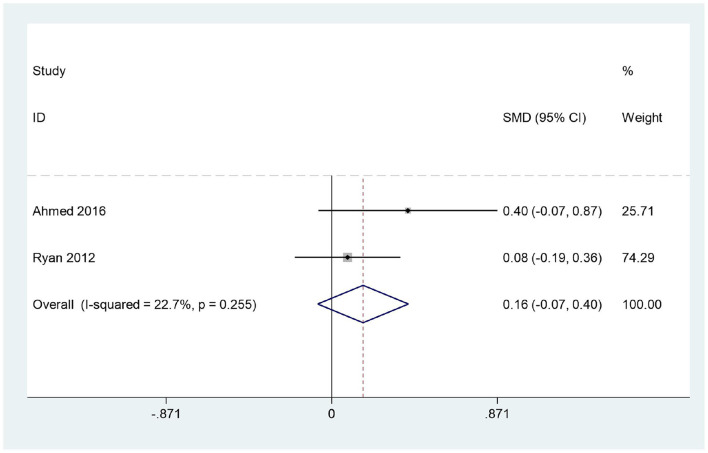
Meta-analysis results and forest plot of digital health intervention on asthma control assessed by mini-AQLQ at 6 months.

**Figure 17:**
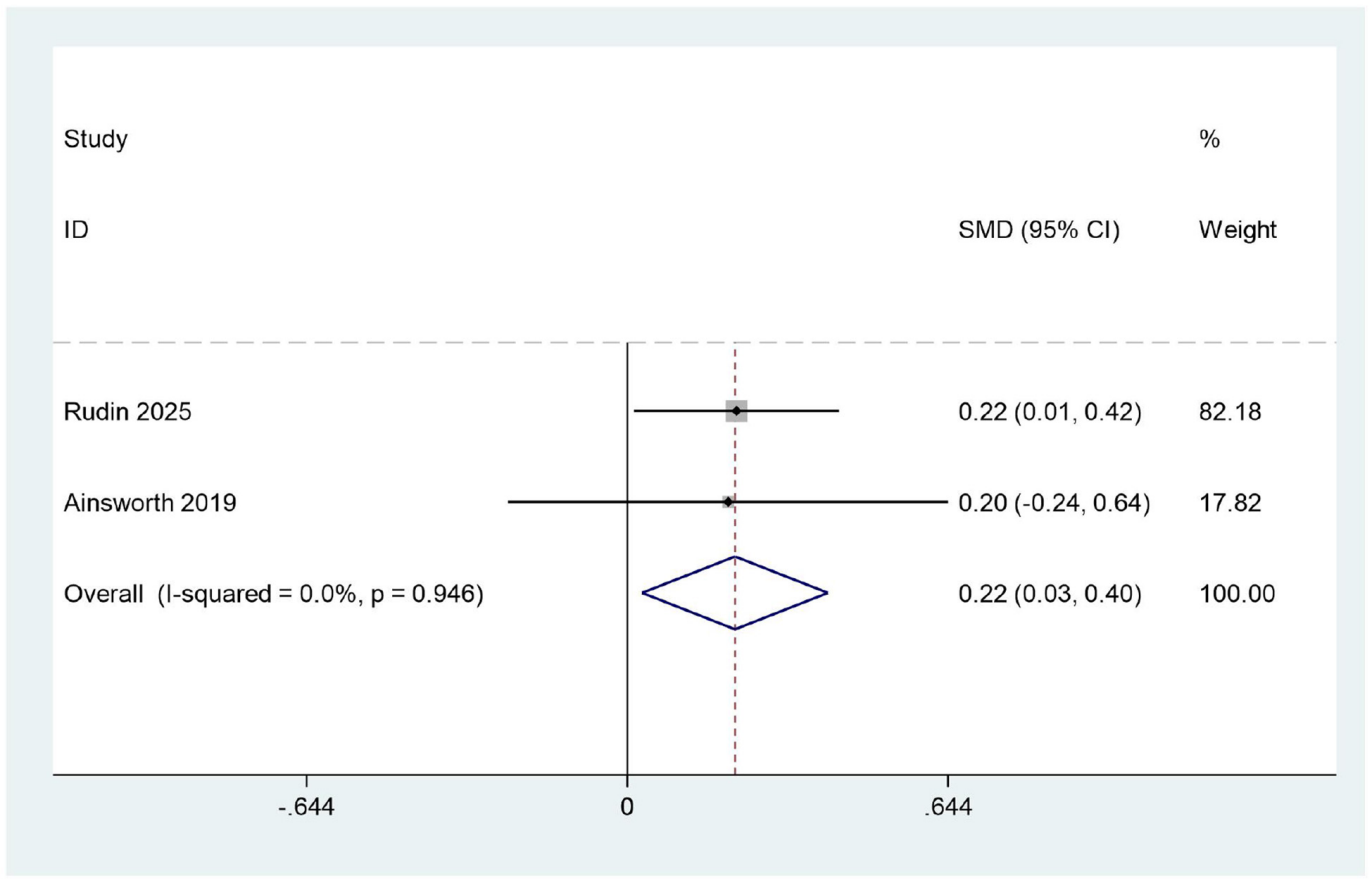
Meta-analysis results and forest plots of digital health intervention on asthma control assessed by mini-AQLQ at 12 months.

A total of 7 studies explored the impact of digital health interventions on mini-AQLQ. Among them, 6 studies provided specific data and were included in the meta-analysis. There was no statistical heterogeneity (*I*^2^ = 0.0%). Using a fixed-effect model, the results showed that the mini-AQLQ score was significantly improved after intervention [SMD = 0.20, 95% CI: 0.06 to 0.34, *P* = 0.005; Figure 14 (16, 18, 23, 25–27)]. Sensitivity analysis confirmed that the mini-AQLQ results were robust. Subgroup analysis showed that there was no significant heterogeneity in the mini-AQLQ score analysis of 4 studies included in the 3-month subgroup (*I*^2^ = 0.0%). The fixed effect model showed that the score was significantly improved [SMD = 0.31, 95% CI: 0.04 to 0.57, *P* = 0.024; Figure 15 (23, 25, 26, 33)]. The 6-month subgroup analysis included 2 studies with low heterogeneity (*I*^2^ = 22.7%). The fixed effect model showed that the intervention effect was not statistically significant [SMD = 0.17, 95% CI: −0.07 to 0.40, *P* = 0.176; Figure 16 (27, 33)]. Two studies were included in the 12-month follow-up to evaluate the 12-month mini-AQLQ score, with no heterogeneity (*I*^2^ = 0.0%). The fixed effect model showed a significant improvement in the score [SMD = 0.22, 95% CI: 0.03 to 0.40, *P* = 0.023; Figure 17 (18, 25)].

##### Pediatric Asthma Quality of Life Questionnaire (PAQLQ)

**Figure 18:**
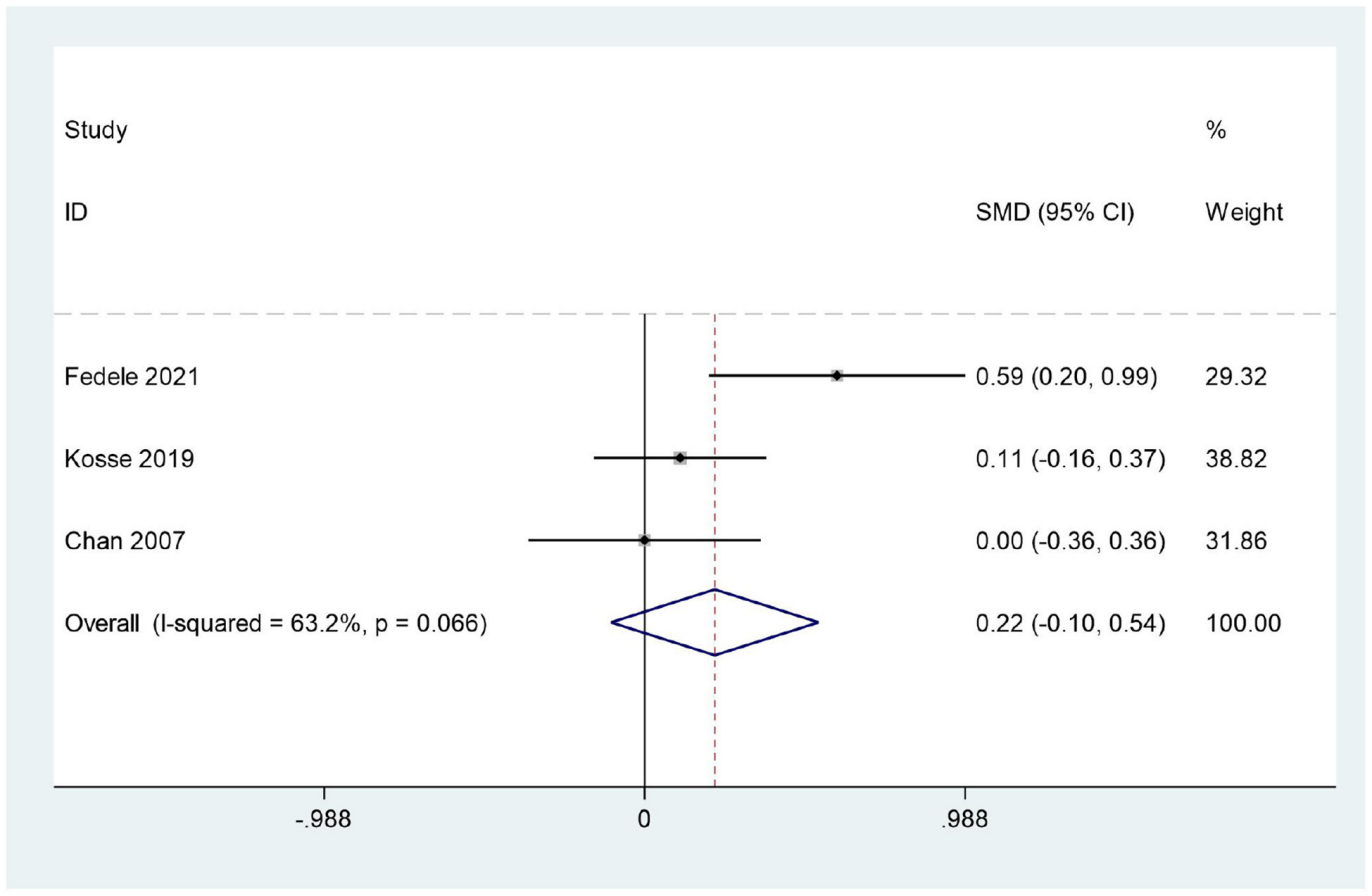
Meta-analysis results and forest plots of digital health intervention on the quality of life of asthma assessed by PAQLQ. Weights are from random effects analysis.

Three studies were included in the PAQLQ analysis, and there was moderate heterogeneity (*I*^2^ = 63.2%). The random effect model showed that the intervention effect was not statistically significant [SMD = 0.22, 95% CI: −0.10 to 0.54, *P* = 0.186; Figure 18 (15, 20, 30)]. Sensitivity analysis showed that Fedele2021 (15) was the main source of heterogeneity. After excluding the study, the heterogeneity of the remaining studies disappeared, but the effect size became smaller and still not significant, indicating that the PAQLQ results were less robust. Due to insufficient data, no subgroup analysis was performed.

#### Functional outcomes

**Figure 19:**
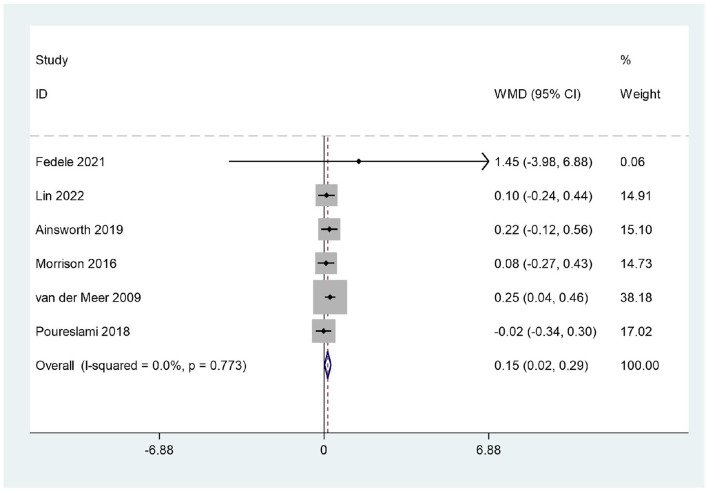
Meta-analysis results and forest plots of digital health intervention on FEV_1_.

**Figure 20:**
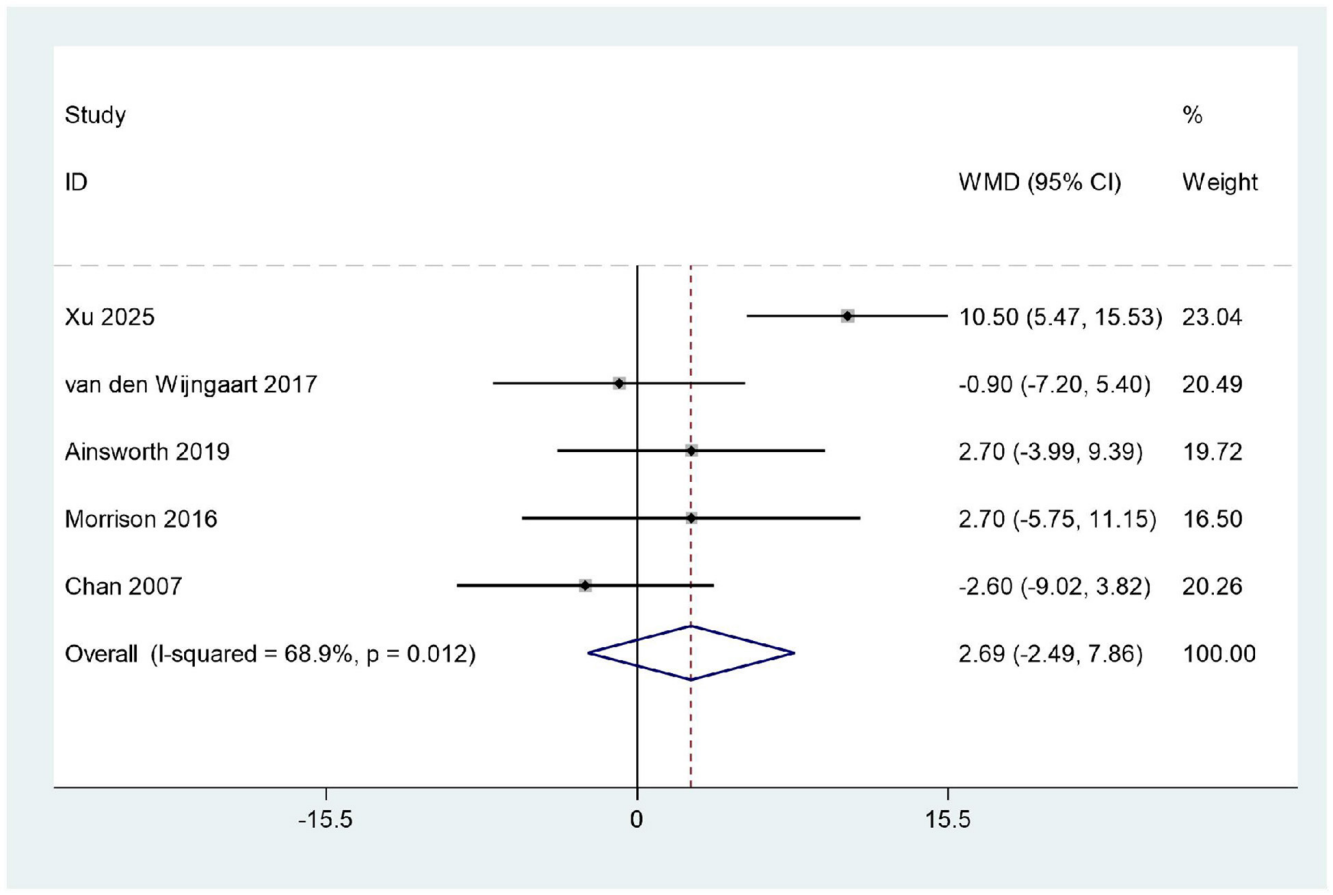
Meta-analysis results and forest plots of digital health intervention on FEV_1_% predicted. Weights are from random effects analysis.

**Figure 21:**
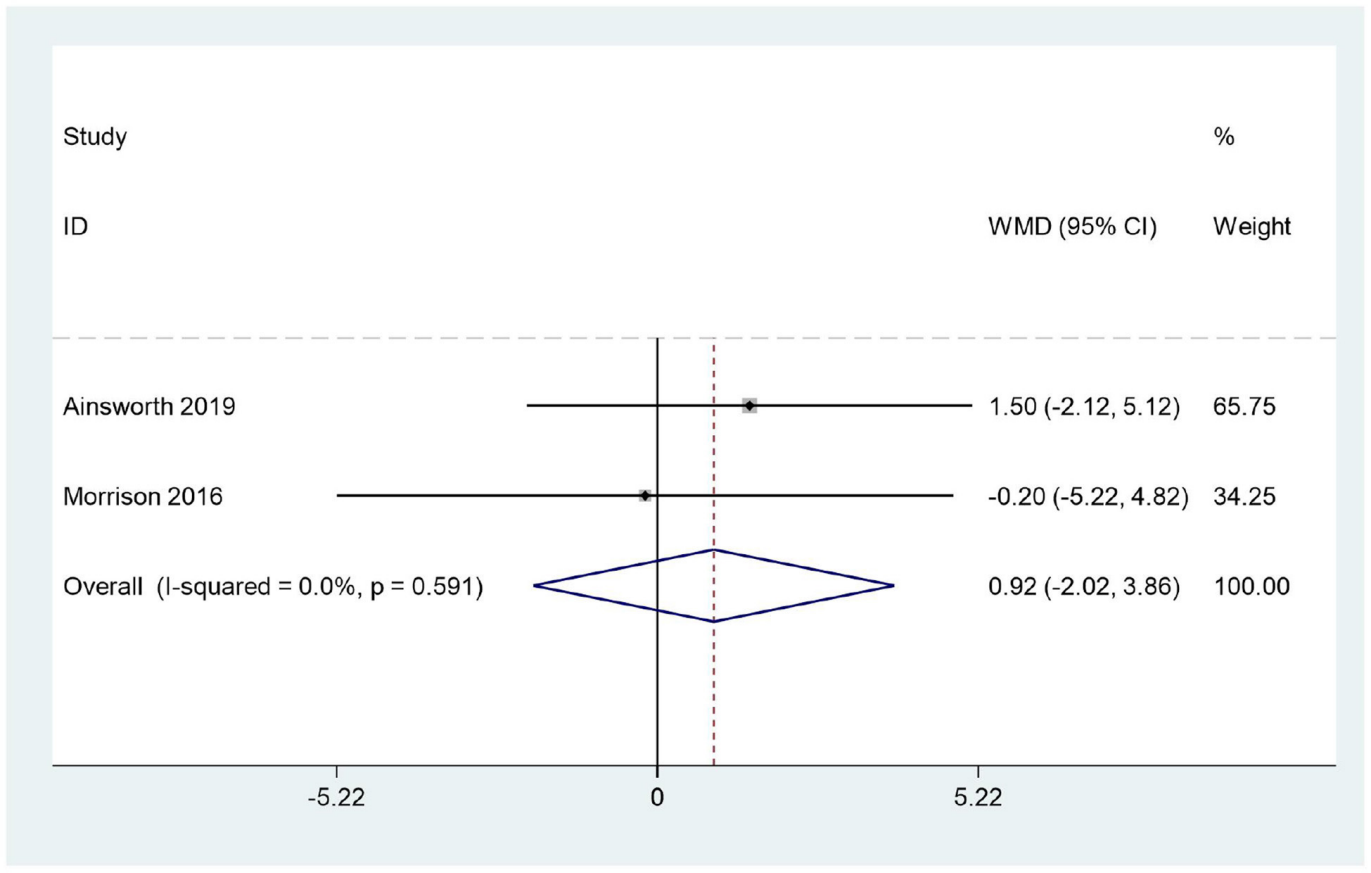
Meta-analysis results and forest plot of digital health intervention on FEV_1_/FVC.

This study systematically evaluated the effect of digital health intervention on improving lung function in patients with asthma. A total of 6 studies reported the effect of interventions on FEV_1_. Meta-analysis of the fixed effect model showed that compared with the control group, the intervention measures could significantly but slightly improve the patient's FEV_1_ [WMD = 0.15, 95% CI: 0.02 to 0.29, *P* = 0.024; Figure 19 (15, 16, 25, 26, 28, 34)]. There was no heterogeneity among the included studies (*I*^2^ = 0.0%), indicating that the results had good consistency. Sensitivity analysis demonstrated that the modest improvement effect of FEV_1_ was generally stable. There was a high degree of heterogeneity among the five studies of FEV_1_% predicted value (*I*^2^ = 68.9%). Random effect model analysis showed that digital health intervention had no significant improvement effect on FEV_1_% predicted value [WMD = 2.69, 95% CI: −2.49 to 7.86, *P* = 0.309; Figure 20 (20, 25, 26, 31, 32)]. Sensitivity analysis showed that the results were less stable, especially affected by Xu et al. (32) study. The fixed effect model of two studies on FEV_1_/FVC showed that the intervention effect was not statistically significant [WMD = 0.92, 95% CI: −2.02 to 3.86, *P* = 0.541; Figure 21 (25, 26)], and there was no heterogeneity (*I*^2^ = 0.0%). Due to the limited number of included studies, the stability of the results was insufficient.

#### Health care use indicators

**Figure 22:**
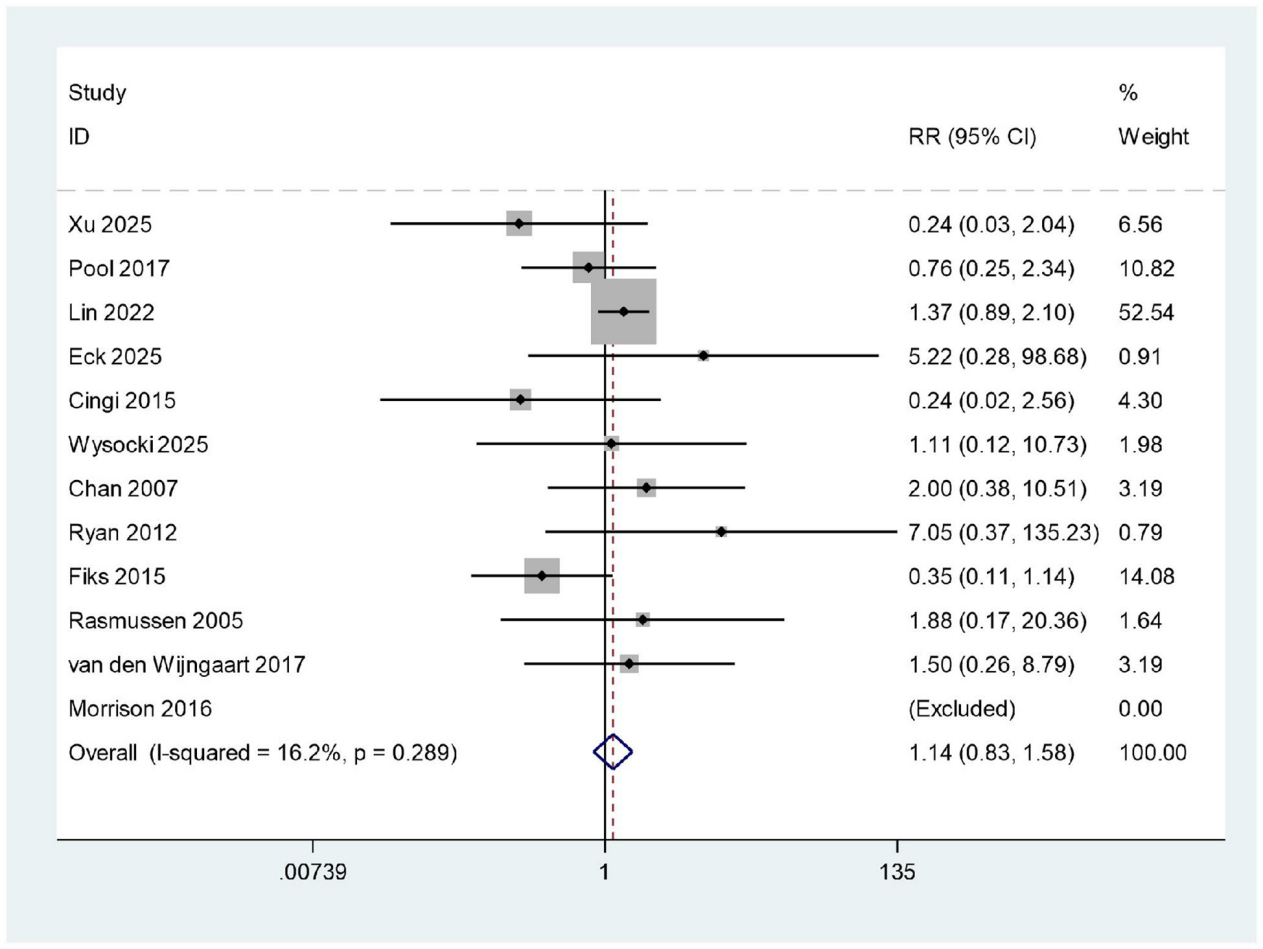
Meta-analysis results and forest map of the impact of digital health intervention on asthma emergency visits.

**Figure 23:**
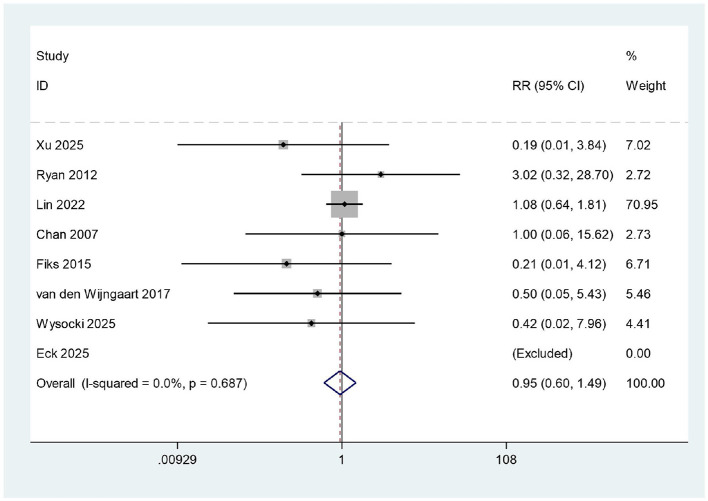
Meta-analysis results and forest map of the impact of digital health intervention on asthma hospitalization.

The medical use indicators include the number of emergency visits and the number of hospitalizations. The overall effect showed that digital health management interventions failed to effectively reduce medical use indicators. The fixed effect model analysis of the number of emergency visits showed that there was no significant difference in the risk of emergency visits between the digital health intervention group and the control group [RR = 1.14, 95% CI: 0.83 to 1.58, *P* = 0.42; *I*^2^ = 16.2%; Figure 22 (16, 19–21, 23, 26, 27, 31, 32, 35, 36, 38)]. Sensitivity analysis showed that the RR value ranged from 0.90 to 1.27, and the results were relatively stable. Funnel plot (Supplementary File 2) and Egger's test showed no significant small study effect (*P* = 0.381), indicating that the possibility of publication bias was small. The fixed effect model analysis of the number of hospitalizations also showed that there was no significant difference in the risk of hospitalization between the two groups (RR = 0.95, 95% CI: 0.60 to 1.49, *P* = 0.818), and there was no heterogeneity between the studies [*I*^2^ = 0.0%; Figure 23 (16, 20, 21, 23, 27, 31, 32, 36)]. Sensitivity analysis showed that the combined RR values after itemized exclusion studies varied between 0.63 and 1.01, and the results were stable.

### Evidence quality evaluation

Due to the limitations of the blind method, asthma control, quality of life and FEV_1_ were rated as having “moderate quality.” Due to the small sample size, wide confidence interval, in some cases crossed the no-effect line, FEV_1_/FVC was rated as very low, while the rest was rated as low quality, which limits the confidence of the estimated effect, and it is recommended that the results should be interpreted with caution. See Supplementary File 2.

## Discussion

### Main findings

This study synthesized data from 24 RCTs involving 5,155 participants to assess the efficacy of digital health interventions for asthma, primarily focusing on self-management tools like self-monitoring, medication reminders, and health information dissemination. While some trials engaged healthcare professionals in the intervention, few addressed strategies for sustaining compliance over time. The findings indicate that digital health interventions significantly enhance patient-reported asthma control and quality of life. Nonetheless, the impact on objective lung function measures is modest, and the reduction in emergency visits and hospitalizations remains elusive.

The level of asthma control was evaluated by ACT and ACQ scores. The results of meta-analysis showed that digital health intervention significantly improved ACT and ACQ scores. The results of the two indicators confirmed each other and formed a consistent conclusion, confirming the positive role of digital health intervention in asthma management. This finding is consistent with previous research results. For example, Xiao et al. (39) found that digital health intervention improved asthma control and compliance. This study further strengthened the clinical value of digital health intervention through the joint verification of the two indicators. Although the improvement in ACT and ACQ scores observed in this meta-analysis was statistically significant, its clinical significance should be interpreted cautiously in light of the minimum clinically important difference (MCID). Taking the ACT as an example, upon conversion of the SMD to the original score, the improvement margin is approximately 1.6 points, falling below the MCID. This suggests that, although improvements in patient-reported outcomes (PROs) were statistically significant across all sensitivity analyses (and not limited to high-risk studies or preliminary trials), their independent clinical significance should be considered limited. Digital health interventions are most effective as adjunctive measures within comprehensive asthma management strategies rather than as standalone treatment modalities. Reductions in perceived symptoms, such as night waking or activity limitation captured by the ACT and ACQ, could contribute to better daily functioning and self-efficacy, serving as a valuable supplementary tool for personalized management, particularly in resource-limited or remote health care settings.

The subgroup analysis of follow-up time revealed the time-dependent difference of intervention effect: the ACT index demonstrates optimal efficacy at 3 months (SMD = 0.45, *P* < 0.001); for ACQ index, the intervention efficacy reached its optimal level at 12-month follow-up (SMD = −0.48, *P* = 0.007), suggesting that the improvement of asthma control reflected by ACQ had a “delayed effect.” This difference may be related to the different evaluation dimensions of the two indicators: ACT focuses more on the short-term changes of patients subjective symptoms (such as wheezing in the past 4 weeks, nocturnal awakening, etc.), while ACQ covers more comprehensive long-term control indicators such as symptoms, emergency drug use, lung function, etc. (40). Its improvement depends on the continuous improvement of self-management ability, suggesting that the improvement of subjective symptoms by digital health intervention is earlier than the improvement of overall control level. In clinical practice, the intervention effect can be dynamically evaluated by combining the two indicators.

Quality of life is an important index to describe the characteristics of patients and evaluate the effect of treatment intervention. AQLQ is a common tool to evaluate the quality of life of asthma patients. This meta-analysis systematically evaluated the effects of digital health intervention on different quality of life scales (AQLQ, mini-AQLQ, PAQLQ) of asthma patients. The results showed obvious scale specificity and time dependence. In the AQLQ score, digital health intervention showed a significant improvement effect, and this effect peaked at 6 months of intervention (SMD = 1.41), and then decreased slightly at 12 months but remained significant (SMD = 0.60), suggesting that digital health intervention has a continuous improvement in the quality of life of adult asthma patients, and the medium-term effect is optimal. This result is highly consistent with the core advantages of digital health intervention. Through real-time symptom monitoring, personalized medication reminders, telemedicine consultation and other functions, patients can quickly master disease management skills in the early stage of intervention, and form stable health behaviors in the middle stage, so as to maximize the improvement of quality of life. The slight attenuation of long-term effects may be related to the decline of intervention compliance or the lack of timely optimization of intervention programs. For mini-AQLQ score, the small but continuous effect size may reflect its value in long-term symptom monitoring. The significant effects in March and December showed that short-term intervention can quickly relieve symptoms, while long-term intervention can maintain the effect through the development of behavioral habits. June may be the “bottleneck period” of patient compliance, and it is necessary to improve the sustainability through program optimization. Notably, no significant improvement was observed in the PAQLQ. Sensitivity analysis showed that the Fedele et al. (15) study was the main source of heterogeneity, but there was no significant difference in the results after excluding the study. This may be due to the special needs of the child population, and the management of asthma in children is highly dependent on the involvement of guardians. In addition, the small number of included studies (*n* = 3) led to insufficient statistical power, and subgroup analysis could not be performed according to age or disease severity. However, when the SMD of AQLQ was converted to the original score, the improvement was about 0.4 points, slightly lower than that of MCID (0.5 points), but may indicate a slight relief of symptoms, such as dyspnea and cough. This has practical significance for daily functional activities, especially for patients with heavy symptom burden. Although these improvements do not directly translate into significant improvements in the overall quality of life, they can help reduce the risk of acute exacerbation or delay disease progression and can be used as an important auxiliary tool for personalized management.

Pulmonary ventilation function index FEV_1_ reflects the severity of airway obstruction, which is the most commonly used evaluation index to objectively judge the condition of asthma. FEV_1_ was slightly but significantly improved, and FEV_1_% predicted value and FEV_1_/FVC index were not significantly improved, which was consistent with the results of previous studies (41). This clearly shows that the main mechanism of digital health intervention may be to optimize behavior patterns, improve medication compliance and self-efficacy, rather than directly reverse or significantly improve the pathophysiological state of the airway.

There was no significant effect on the number of emergency visits and the number of hospitalizations, possibly due to the relatively stable overall condition of the patients in the study and the low incidence of baseline events, leading to inadequate statistical power. To translate enhancements in subjective symptoms into reduced acute episodes necessitating clinical intervention, more robust interventions closely aligned with the healthcare system's response and extended follow-up periods to accrue an adequate number of events are essential.

### Relationship with previous literature

Previously published systematic reviews have confirmed the beneficial impact of digital health interventions on various chronic diseases, including chronic obstructive pulmonary disease (42), cardiovascular disease prevention (43), and weight management in children and adolescents (44). Existing reviews on asthma have predominantly focused on specific intervention modules or technical approaches, such as childhood asthma management (45), the effect of remote monitoring on clinical outcomes (12), the utilization of two-way asynchronous communication between healthcare providers and patients in asthma care (46, 47), the cost-benefit analysis of digital health intervention (48), strategies to enhance medication adherence (41), electronic monitoring of inhaled glucocorticoids (49), the impact of telemedicine on quality of life (50) and the effectiveness of symptom control (51). However, these studies often concentrate on individual functions, like remote monitoring, or specific technical pathways, neglecting the comprehensive integration of electronic health records, wearable devices, and intelligent data analysis platforms within a unified health management system. Therefore, this systematic review aims to broaden the scope of prior research by incorporating recent evidence featuring multi-module integration aspects (e.g., health education, behavioral motivation, social support, and remote monitoring) to provide a more comprehensive assessment of the overall effectiveness and implementation potential of digital health interventions in asthma management.

## Limitations

This study has several limitations that warrant consideration. First, the majority of included outcomes relied on unblinded self-reports, which are susceptible to recall and social desirability bias. Second, despite conducting subgroup and sensitivity analyses, substantial heterogeneity persisted; this may be attributable to the broad diversity of intervention components and the inconsistent application of behavioral change theories, which could not be fully disentangled. Third, potential publication bias cannot be excluded, as formal assessment was not feasible for all outcomes due to the limited number of studies per comparison. Fourth, pediatric-specific analyses were constrained by the small number of child-focused trials, limiting the generalizability of findings to pediatric populations. Fifth, the meta-analyses for healthcare utilization outcomes were likely underpowered to detect significant differences. Sixth, long-term effects beyond 12 months remain uncertain owing to insufficient follow-up data. Finally, some prespecified subgroup analyses could not be performed because of an inadequate number of studies.

Finally, the systematic literature search was completed on December 31, 2025. Given the rapid pace of research in digital health, it is possible that relevant randomized controlled trials published during this interval were not included. We have transparently reported the search timeframe, and as the evidence base continues to expand, a future update of this review will be warranted to incorporate newly available studies.

### Implications for medical practice and further research

Digital health interventions, particularly those used continuously for over three months, can improve patient-reported asthma control and quality of life. However, these subjective improvements have not been accompanied by measurable gains in lung function or reductions in acute care utilization. Clinical programmes may therefore prioritize patient-centred outcomes, but they should avoid overpromising on the reduction of exacerbations or hospitalizations without stronger supporting evidence. The gap between improved patient-reported outcomes (PROs) and unchanged physiological or utilization endpoints suggests that future interventions need stronger adherence mechanisms, clinician escalation pathways, and integration with objective monitoring (such as remote spirometry or AI-assisted pulmonary function analysis) to translate perceived benefits into clinically meaningful hard endpoints.

In the present study, pre-planned subgroup analyses were conducted by follow-up duration (3, 6, and 12 months) to explore temporal effect patterns. Because many digital interventions are complex and multicomponent, and because the level of reporting detail varies considerably across studies, we were unable to perform subgroup analyses based on specific intervention components. Future research should adopt a standardized reporting framework that thoroughly describes intervention components, theoretical foundations, and adherence strategies. This would enable component-specific subgroup analyses or network meta-analyses to disentangle the active ingredients of digital interventions. Trials with larger samples, longer follow-up, and inclusion of high-risk populations are also needed to evaluate whether digital interventions can reduce acute episodes and healthcare costs. Individual patient data meta-analysis is recommended to further investigate sources of heterogeneity, determine optimal intervention duration, and identify which patient subgroups benefit most.

## Conclusion

Our analysis indicates that digital health interventions hold promise for enhancing self-reported control levels and quality of life across certain dimensions for individuals with asthma. Nevertheless, their direct influence on objective lung function metrics and healthcare resource utilization is constrained. It should also be noted that the digital interventions included in this review exhibited considerable heterogeneity in their components, ranging from simple medication reminders to comprehensive programs incorporating non-pharmacological elements such as breathing exercises and behavioral training. This variation may have contributed to the observed effects, and we have explicitly pointed out this limitation in the manuscript by acknowledging that the inability to conduct subgroup analyses based on specific intervention components restricts the granularity of the findings. Future endeavors should focus on crafting more tailored digital remedies that are closely intertwined with the clinical care framework and capable of sustaining extended user engagement. By conducting more stringent, enduring investigations, we can substantiate their efficacy in enhancing critical clinical outcomes for patients and refining the distribution of medical resources.

## Data Availability

The original contributions presented in the study are included in the article/Supplementary material, further inquiries can be directed to the corresponding author.
