# Supplementary material for: Effectiveness of digital health interventions for asthma: a systematic review and meta-analysis
Source: Front Public Health. 2026 Aug 5;14:1910816. doi: 10.3389/fpubh.2026.1910816 (PMC13486186; doi:10.3389/fpubh.2026.1910816)
Supplement: Supplementary file 2 [file Data_Sheet_2.docx]

**Supplementary Table 1:** Details of the Literature Search Strategy

（1） **PubMed （108 results）**

| **Search** | **Search Terms/Strategy** |
| --- | --- |
| #1 | ((((((((((asthma[MeSH Terms]) OR (Asthmas[Title/Abstract])) OR (Asthma, Bronchial[Title/Abstract])) OR (Bronchial Asthma[Title/Abstract])) OR (asthma bronchiale[Title/Abstract])) OR (asthma pulmonale[Title/Abstract])) OR (asthmatic[Title/Abstract])) OR (asthmatic subject[Title/Abstract])) OR (bronchus asthma[Title/Abstract])) OR (chronic asthma[Title/Abstract])) OR (lung allergy[Title/Abstract]) |
| #2 | (((((((((((((((((((telemedicine[MeSH Terms]) OR (Tele[Title/Abstract])) OR (Mobile Health[Title/Abstract])) OR (mHealth[Title/Abstract])) OR (eHealth[Title/Abstract])) OR (biosensor[Title/Abstract])) OR (remote monitoring[Title/Abstract])) OR (Smartphone[MeSH Terms])) OR (Smartphone[Title/Abstract])) OR (Phone[Title/Abstract])) OR (Mobile Applications[Title/Abstract])) OR (Mobile Application[Title/Abstract])) OR (App[Title/Abstract])) OR (Internet[MeSH Terms])) OR (Internet[Title/Abstract])) OR (Web[Title/Abstract])) OR (website[Title/Abstract])) OR (digital health[Title/Abstract])) OR (online[Title/Abstract])) OR (information technology[Title/Abstract]) |
| #3 | (((((self-management[MeSH Terms]) OR (self care[Title/Abstract])) OR (self management[Title/Abstract])) OR (self monitoring[Title/Abstract])) OR (self-monitoring[Title/Abstract])) OR (self-care[Title/Abstract]) |
| #4 | (((random* controlled trial[Publication Type]) OR (controlled clinical trial[Publication Type])) OR (random[Title/Abstract])) OR (trial[Title/Abstract]) |
| #5 | #1 AND #2 AND #3 AND #4 |

（2） **Embase （275 results）**

| **Search** | **Search Terms/Strategy** |
| --- | --- |
| #1 | 'Asthma'/exp |
| #2 | 'Asthmas':ab,ti OR 'Asthma, Bronchial':ab,ti OR 'Bronchial Asthma':ab,ti OR 'asthma bronchiale':ab,ti OR 'asthma pulmonale':ab,ti OR 'asthmatic':ab,ti OR 'asthmatic subject':ab,ti OR 'bronchus asthma':ab,ti OR 'chronic asthma':ab,ti OR 'lung allergy':ab,ti |
| #3 | #1 OR #2 |
| #4 | 'telemedicine'/exp |
| #5 | tele:ab,ti OR 'mobile health':ab,ti OR mhealth:ab,ti OR ehealth:ab,ti OR biosensor:ab,ti OR 'remote monitoring':ab,ti |
| #6 | #4 OR #5 |
| #7 | 'smartphone'/exp |
| #8 | smartphone:ab,ti OR phone:ab,ti |
| #9 | #7 OR #8 |
| #10 | 'mobile application'/exp |
| #11 | 'mobile application':ab,ti OR app:ab,ti |
| #12 | #10 OR #11 |
| #13 | 'internet'/exp |
| #14 | internet:ab,ti OR web:ab,ti OR website:ab,ti OR 'digital health':ab,ti OR online:ab,ti OR 'information technology':ab,ti |
| #15 | #13 OR #14 |
| #16 | #6 OR #9 OR #12 OR #15 |
| #17 | 'self care'/exp |
| #18 | 'self management':ab,ti OR 'self monitoring':ab,ti OR 'self care':ab,ti |
| #19 | #17 OR #18 |
| #20 | 'randomized controlled trial'/exp |
| #21 | 'random* controlled trial':ab,ti OR 'controlled clinical trial':ab,ti OR random:ab,ti OR trial:ab,ti |
| #22 | #20 OR #21 |
| #23 | #3 AND #16 AND #19 AND #22 |

（3） **Cochrane Library (CENTRAL) （217 results）**

| **Search** | **Search Terms/Strategy** |
| --- | --- |
| #1 | MeSH descriptor: [Asthma] explode all trees |
| #2 | (Asthmas):ti,ab,kw OR (Asthma, Bronchial):ti,ab,kw OR (Bronchial Asthma):ti,ab,kw OR (asthma bronchiale):ti,ab,kw OR (asthma pulmonale):ti,ab,kw OR (asthmatic):ti,ab,kw OR (asthmatic subject):ti,ab,kw OR (bronchus asthma):ti,ab,kw OR (chronic asthma):ti,ab,kw OR (lung allergy):ti,ab,kw |
| #3 | #1 OR #2 |
| #4 | MeSH descriptor: [Telemedicine] explode all trees |
| #5 | (telemedicine):ti,ab,kw OR (Tele):ti,ab,kw OR (Mobile Health):ti,ab,kw OR (mHealth):ti,ab,kw AND (eHealth):ti,ab,kw |
| #6 | (biosensor):ti,ab,kw OR (remote monitoring):ti,ab,kw |
| #7 | #4 OR #5 OR #6 |
| #8 | MeSH descriptor: [Smartphone] explode all trees |
| #9 | (Smartphone):ti,ab,kw OR (Phone):ti,ab,kw OR (Mobile Applications):ti,ab,kw OR (Mobile Application):ti,ab,kw OR (App):ti,ab,kw |
| #10 | #8 OR #9 |
| #11 | MeSH descriptor: [Internet] explode all trees |
| #12 | (Internet):ti,ab,kw OR (Web):ti,ab,kw OR (website):ti,ab,kw OR (digital health):ti,ab,kw OR (online):ti,ab,kw |
| #13 | (information technology):ti,ab,kw |
| #14 | #11 OR #12 OR #13 |
| #15 | #7 OR #10 OR #14 |
| #16 | MeSH descriptor: [Self-Management] explode all trees |
| #17 | (self-management):ti,ab,kw OR (self care):ti,ab,kw OR (self management):ti,ab,kw OR (self monitoring):ti,ab,kw OR (self-monitoring):ti,ab,kw |
| #18 | (self-care):ti,ab,kw |
| #19 | #16 OR #17OR #18 |
| #20 | MeSH descriptor: [Randomized Controlled Trial] explode all trees |
| #21 | (random* controlled trial):ti,ab,kw OR (controlled clinical trial):ti,ab,kw OR (random):ti,ab,kw OR (trial):ti,ab,kw |
| #22 | #20 OR #21 |
| #23 | #3 AND #15AND #19 AND #22 |

（4） **Web of Science （906 results）**

| **Search** | **Search Terms/Strategy** |
| --- | --- |
| #1 | TS= (Asthma OR Asthmas OR Asthma, Bronchial OR Bronchial Asthma OR asthma bronchiale OR asthma pulmonale OR asthmatic OR asthmatic subject OR bronchus asthma OR chronic asthma OR lung allergy) |
| #2 | TS= (Telemedicine OR Tele* OR Mobile Health OR mHealth OR eHealth OR biosensor OR remote monitoring OR Internet OR Smartphone* OR Phone* OR Mobile Application* OR App OR Web OR website* OR digital health OR online OR information technology) |
| #3 | TS= (self-management OR self care OR self management OR self monitoring OR self-monitoring OR self-care) |
| #4 | TS= (randomized controlled trial OR random* controlled trial OR controlled clinical trial* OR random* OR trial*) |
| #5 | #1 AND #2 AND #3 AND #4 |

（5） **Scopus （341 results）**

| **Search** | **Search Terms/Strategy** |
| --- | --- |
| #1 | Asthma OR Asthmas OR Asthma, Bronchial OR Bronchial Asthma OR asthma bronchiale OR asthma pulmonale OR asthmatic OR asthmatic subject OR bronchus asthma OR chronic asthma OR lung allergy |
| #2 | Telemedicine OR Tele* OR Mobile Health OR mHealth OR eHealth OR biosensor OR remote monitoring OR Internet OR Smartphone* OR Phone* OR Mobile Application* OR App OR Web OR website* OR digital health OR online OR information technology |
| #3 | self-management OR self care OR self management OR self monitoring OR self-monitoring OR self-care |
| #4 | randomized controlled trial OR random* controlled trial OR controlled clinical trial* OR random* OR trial* |
| #5 | #1 AND #2 AND #3 AND #4 |

**Supplementary Table 2:** Summary of Findings.

| **Quality assessment** | | | | | | | **No of patients** | | **Effect** | | **Quality** | **Importance** |
| --- | --- | --- | --- | --- | --- | --- | --- | --- | --- | --- | --- | --- |
|  |  |  |  |  |  |  |  |  |  |  |  |  |
| **No of studies** | **Design** | **Risk of bias** | **Inconsistency** | **Indirectness** | **Imprecision** | **Other considerations** | **digital health intervention** | **usual care** | **Relative (95% CI)** | **Absolute** |  |  |
| **ACT** | | | | | | | | | | | | |
| 11 | randomised trials | serious^1^ | no serious inconsistency | no serious indirectness | no serious imprecision | none | 1238 | 1222 | - | SMD 0.43 higher (00.35 to 0.51 higher) | ⊕⊕⊕O MODERATE | CRITICAL |
| **ACQ** | | | | | | | | | | | | |
| 6 | randomised trials | serious^1^ | no serious inconsistency | no serious indirectness | no serious imprecision | none | 428 | 434 | - | SMD 0.37 lower (0.66 to 0.08 lower) | ⊕⊕⊕O MODERATE | CRITICAL |
| **AQLQ** | | | | | | | | | | | | |
| 4 | randomised trials | serious^1^ | no serious inconsistency | no serious indirectness | no serious imprecision | none | 217 | 208 | - | SMD 0.98 higher (00.49 to 1.47 higher) | ⊕⊕⊕O MODERATE | CRITICAL |
| **mini-AQLQ** | | | | | | | | | | | | |
| 6 | randomised trials | serious^1^ | no serious inconsistency | no serious indirectness | no serious imprecision | none | 365 | 428 | - | SMD 0.20 higher (0.06 to 0.34 higher) | ⊕⊕⊕O MODERATE | CRITICAL |
| **PAQLQ** | | | | | | | | | | | | |
| 3 | randomised trials | serious^1^ | no serious inconsistency | no serious indirectness | serious^2^ | none | 200 | 257 | - | SMD 0.22 higher (0.10 lower to 0.54 higher) | ⊕⊕OO LOW | CRITICAL |
| **FEV1** | | | | | | | | | | | | |
| 6 | randomised trials | serious^1^ | no serious inconsistency | no serious indirectness | no serious imprecision | none | 315 | 322 | - | SMD 0.18 higher (0.02 to 0.33 higher) | ⊕⊕⊕O MODERATE | CRITICAL |
| **FEV1 % predicted** | | | | | | | | | | | | |
| 5 | randomised trials | serious^1^ | no serious inconsistency | no serious indirectness | serious^2^ | none | 229 | 208 | - | MD 2.69 higher (2.49 lower to 7.86 higher) | ⊕⊕OO LOW | CRITICAL |
| **FEV1 / FVC** | | | | | | | | | | | | |
| 2 | randomised trials | serious^1^ | no serious inconsistency | no serious indirectness | very serious^3^ | none | 56 | 69 | - | MD 0.92 higher (2.12 lower to 5.12 higher) | ⊕OOO VERY LOW | CRITICAL |
| **emergency visits** | | | | | | | | | | | | |
| 12 | randomised trials | serious^1^ | no serious inconsistency | no serious indirectness | serious^2^ | none | 71/1239  (5.7%) | 62/1213  (5.1%) | RR 1.14 (0.83 to 1.58) | 7 more per 1000 (from 9 fewer to 30 more) | ⊕⊕OO LOW | CRITICAL |
|  |  |  |  |  |  |  |  | 75% |  | 105 more per 1000 (from 128 fewer to 435 more) |  |  |
| **hospitalization** | | | | | | | | | | | | |
| 8 | randomised trials | serious^1^ | no serious inconsistency | no serious indirectness | serious^2^ | none | 33/916  (3.6%) | 36/911  (4%) | RR 0.95 (0.60 to 1.49) | 2 fewer per 1000 (from 16 fewer to 19 more) | ⊕⊕OO LOW | CRITICAL |
|  |  |  |  |  |  |  |  | 75% |  | 38 fewer per 1000 (from 300 fewer to 368 more) |  |  |

^1^ The allocation of concealment and blinding has limitations.
^2^ above the equipotential line
^3^ above the isocline and with small sample size

**Supplementary Table 3: Sensitivity Analysis Excluding High-Risk-of-Bias Trials and Pilot Studies**

| **PRO measure** | **Analysis** | **k** | **SMD (95% CI)** | **P value** | **I²** |
| --- | --- | --- | --- | --- | --- |
| ACT | Primary analysis (all 11 studies) | 11 | 0.43 (0.35, 0.51) | <0.001 | 0.474 |
|  | Excluding 2 high-risk-of-bias trials | 9 | 0.44 (0.30, 0.58) | <0.001 | 0.518 |
|  | Excluding 1 pilot study | 10 | 0.42 (0.34, 0.50) | <0.001 | 0.409 |
|  | Excluding all high-risk and pilot studies | 8 | 0.41 (0.33, 0.50) | <0.001 | 0.448 |
| ACQ | Primary analysis (all 6 studies) | 6 | −0.37 (−0.66, −0.08) | 0.013 | 0.766 |
|  | Excluding 1 pilot study | 5 | −0.38 (−0.70, −0.05) | 0.025 | 0.813 |
| AQLQ | Primary analysis (all 4 studies) | 4 | 0.98 (0.49, 1.47) | <0.001 | 0.803 |
|  | No exclusions required | — | — | — | — |
| Mini-AQLQ | Primary analysis (all 6 studies) | 6 | 0.20 (0.06, 0.34) | 0.005 | 0 |
|  | Excluding 1 high-risk-of-bias trial | 5 | 0.20 (0.06, 0.34) | 0.006 | 0 |
|  | Excluding 1 pilot study | 5 | 0.20 (0.05, 0.34) | 0.008 | 0 |
|  | Excluding high-risk and pilot studies | 4 | 0.20 (0.05, 0.34) | 0.009 | 0 |

Abbreviations: PRO: patient-reported outcome; k: number of studies; SMD: standardized mean difference; CI: confidence interval.

Notes: High-risk-of-bias trials were assessed using the Cochrane Risk of Bias 2 tool. Pilot studies were defined as those explicitly described as pilot/feasibility trials. For ACQ, no high-risk-of-bias trials were identified among the included studies. For AQLQ, none of the included studies were rated as high risk of bias or designed as pilot studies; therefore, no sensitivity exclusions were performed. Dashes (—) indicate that the metric is not applicable.

**Supplementary Fig. 1** Evaluation of publication bias using funnel plots for ACT.

**Supplementary Fig. 2** Evaluation of publication bias using funnel plots for the number of emergency visits.
